# Sex- and menopause-specific inverse associations between metabolic dysfunction–associated steatotic liver disease and serum lipoprotein(a) concentrations: evidence from SHIP and UK Biobank

**DOI:** 10.1186/s12933-026-03289-8

**Published:** 2026-07-12

**Authors:** Júlia Galbiati de Souza, Till Ittermann, Nicole Werner, Sabine Schipf, Nele Friedrich, Matthias Nauck, Marcio Hiroshi Miname, Fábio Fernandes, Raul Dias Santos, Jelena-Rima Ghadri, Davide Di Vece, Martin Bahls, Giovanni Targher, Elisabeth Steinhagen-Thiessen, Christian Templin, Nágila Raquel Teixeira Damasceno, Marcello Ricardo Paulista Markus

**Affiliations:** 1 Postgraduate Program in Cardiology, Heart Institute (InCor), University of São Paulo Medical School, São Paulo, Brazil; 2 Department of Study of Health in Pomerania/Clinical-Epidemiological Research, Institute for Community Medicine, University Medicine Greifswald, Greifswald, Germany; 3 German Centre for Cardiovascular Research (DZHK), Partner Site North, Greifswald, Germany; 4 German Center for Diabetes Research (DZD), Greifswald, Germany; 5 Institute of Clinical Chemistry and Laboratory Medicine, University Medicine Greifswald, Greifswald, Germany; 6 Lipid Clinic, Heart Institute (InCor), University of São Paulo Medical School, São Paulo, Brazil; 7 Cardiomyopathy Unit, Heart Institute (InCor), University of São Paulo Medical School, São Paulo, Brazil; 8 Academic Research Organization, Hospital Israelita Albert Einstein, São Paulo, Brazil; 9 Department of Internal Medicine B, University Medicine Greifswald, Greifswald, Germany; 10 First Clinic of Internal Medicine, Department of Internal Medicine, University of Genoa, Genoa, Italy; 11 Metabolic Diseases Research Unit, IRCCS Sacro Cuore - Don Calabria Hospital, Negrar di Valpolicella, Italy; 12 Department of Medicine, University of Verona, Verona, Italy; 13 Lipid Clinic at the Interdisciplinary Metabolism Center, Charité – University Medicine Berlin, Berlin, Germany; 14 Institute of Clinical Chemistry and Laboratory Medicine, University Medicine Rostock, Rostock, Germany; 15 Department of Nutrition, School of Public Health, University of São Paulo, São Paulo, Brazil

**Keywords:** Arterial stiffness, Concentric remodelling, Diabetes mellitus, Insulin resistance, Prediabetes

## Abstract

**Background:**

The association between metabolic dysfunction-associated steatotic liver disease (MASLD) and serum lipoprotein(a) (Lp[a]) levels remains controversial, with no sex- or menopausal status-stratified analyses. We aimed to analyse the associations between MASLD, liver fat content (LFC) and transaminases with Lp(a) concentrations stratified by sex and menopausal status.

**Methods:**

We analysed data from 3825 individuals (1961 females; 51.3%) aged 32 to 70 years from the SHIP-START-0 cohort, and from 28,504 individuals (14,926 females; 52.4%) aged 38 to 72 years from the UK Biobank cohort. MASLD was determined by liver ultrasound examinations in SHIP-STAR-0 and by magnetic resonance imaging in UK Biobank. We examined sex- and menopausal status-specific associations of MASLD, LFC, alanine aminotransferase (ALT), aspartate aminotransferase (AST), and gamma-glutamyltransferase (GGT) with lipoprotein(a) [Lp(a)] concentrations, after adjustment for age, body mass index, haemoglobin A1c, glucose-lowering medication use, hypertension, smoking, and alcohol consumption.

**Results:**

In SHIP-START-0, MASLD, higher ALT, AST, and GGT levels were independently associated with lower Lp(a) concentrations only in males but not in females. However, after stratification by menopausal status, higher ALT levels were associated with lower Lp(a) concentrations in postmenopausal females, but not in premenopausal females. In UK Biobank, MASLD severity (moderate and severe), higher LFC, and higher serum transaminase levels were independently associated with lower Lp(a) concentrations in males. Higher LFC was also associated with lower Lp(a) concentrations in females, but serum liver enzymes were not. After stratification by menopausal status, MASLD severity and higher LFC were associated with lower Lp(a) concentrations in postmenopausal females, but not in premenopausal females. Males with MASLD had 24% and 9% lower Lp(a) concentrations than those without MASLD in SHIP-START-0 and UK Biobank.

**Conclusions:**

Our findings from two large community-based studies show that MASLD and higher LFC and ALT values were independently associated with lower Lp(a) concentrations in postmenopausal females and males. Future studies are needed to determine whether the cardiovascular risk in patients with MASLD remains elevated even when Lp(a) concentrations are reduced and whether these patients require more intensive treatment to further reduce Lp(a) concentrations below the currently recommended cut-off values.

**Graphical abstract:**

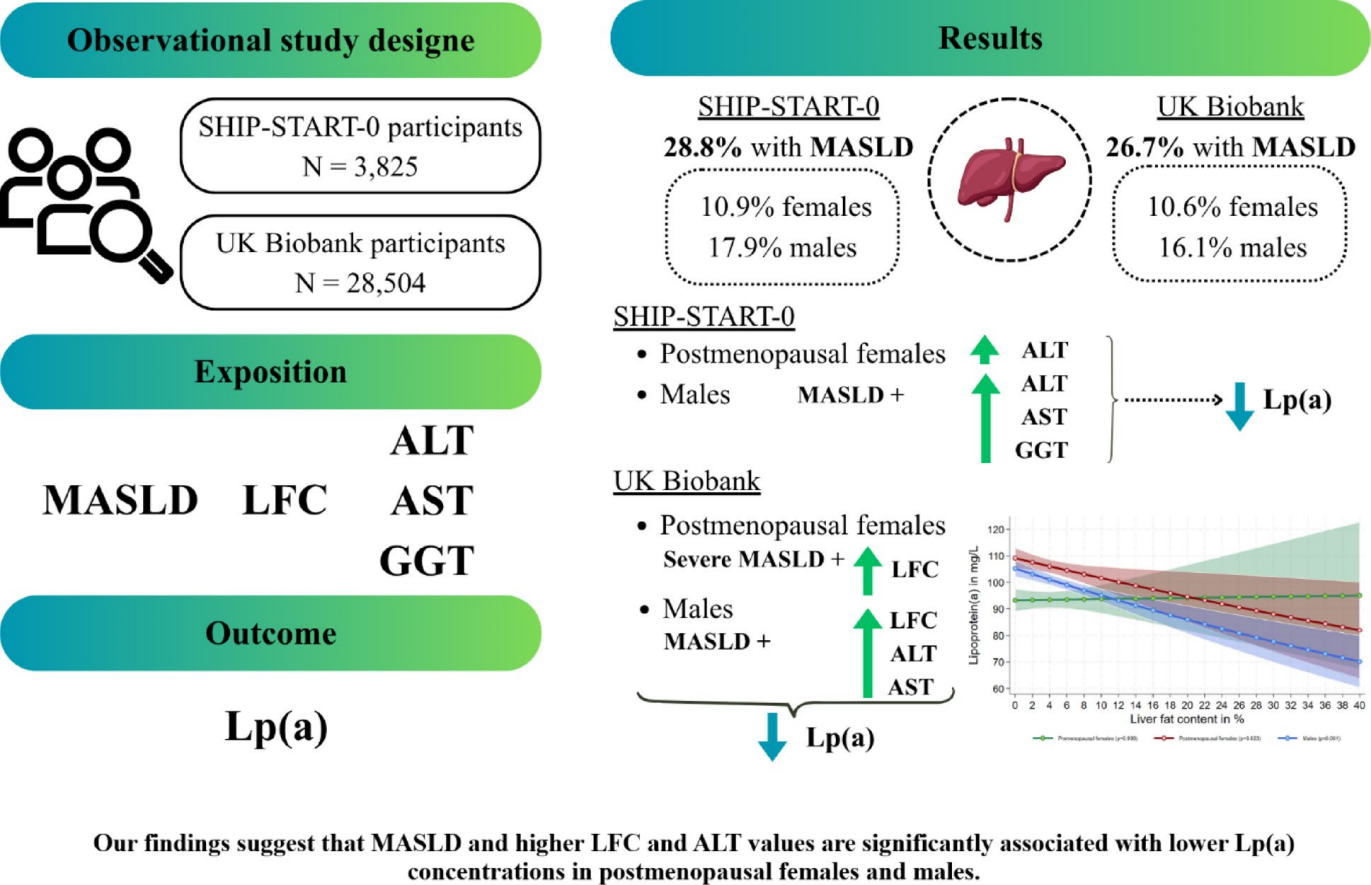

**Supplementary Information:**

The online version contains supplementary material available at 10.1186/s12933-026-03289-8.

## Research Insights

**What is currently known about this topic?**

- Metabolic dysfunction-associated steatotic liver disease (MASLD) may compromise several physiological liver functions. Lipoprotein(a) (Lp[a]) is a cholesterol-rich low-density lipoprotein-like particle synthesized by hepatocytes.

**What is the main research question?**

- Are there specific associations by sex and menopausal status between MASLD and circulating Lp(a) concentrations?

**What’s new?**

- This study showed that MASLD and higher liver fat content and alanine aminotransferase were independently associated with lower Lp(a) concentrations in postmenopausal females and males.

**How might this study influence clinical practice?**

- It may be considered that Lp(a) concentrations might change over the lifetime under metabolic dysfunction-associated conditions.

## Background

Cardiovascular diseases (CVD) are the leading cause of mortality worldwide [1]. The prevalence of CVD has grown significantly in recent years, from approximately 271 million individuals in 1990 to ~ 523 million in 2019 [2]. Likewise, CVD mortality has increased from ~ 12.1 million in 1990 to ~ 18.6 million in 2019 [2]. Epidemiological studies and pathophysiological findings report significant associations between dyslipidemia, atherosclerosis, and increased CVD risk [3, 4]. In addition to the established association between increased low-density lipoprotein cholesterol (LDL-C) concentrations and CVD risk, higher lipoprotein(a) [Lp(a)] levels are also considered an emerging CVD risk factor [5–7]. Growing evidence shows that higher Lp(a) concentrations in the general adult population are more prevalent than initially estimated, and this prevalence is likely underestimated, especially in low-income countries where access to at least one life serum Lp(a) measurement is limited.

Lp(a) is a cholesterol-rich LDL-like particle synthesized by hepatocytes that contains an additional apolipoprotein, apolipoprotein(a) [apo(a)] covalently attached to apolipoprotein B-100 (apoB) [8]. Circulating levels of Lp(a) are mostly genetically determined [9], and remain relatively stable throughout life (except in postmenopausal females and inflammatory conditions) [10], and are less influenced by age, sex, or body weight than other classes of lipoproteins [11, 12]. Menopause is associated with an approximately 10–30% increase in serum Lp(a) levels, which decrease with oestrogen replacement therapy [13]. 

On the other hand, our group has published two studies [14, 15] showing a strong association between metabolic dysfunctions and Lp(a) concentrations. In the first study, involving 7443 participants from two independent population datasets, we observed that higher plasma glucose levels were associated with lower Lp(a) concentrations in males, but not in females [14]. In the second study, also using data from two independent cohorts, we found that postmenopausal females and males with metabolic syndrome had significantly lower Lp(a) levels than their counterparts without metabolic syndrome. Conversely, in premenopausal females, the result was the opposite, showing that premenopausal females with metabolic syndrome had higher Lp(a) concentrations than their counterparts without metabolic syndrome [15]. 

Since the liver is the exclusive site of Lp(a) synthesis and clearance, the profound metabolic and inflammatory remodelling inherent to metabolic dysfunction-associated steatotic liver disease (MASLD), formerly termed non-alcoholic fatty liver disease (NAFLD), may directly alter Lp(a) homeostasis. Investigating this association is crucial because hepatic lipid dysregulation and low-grade inflammation could modulate Lp(a) expression, potentially compounding the residual cardiovascular risk already present in MASLD patients. Currently, MASLD affects up to 35–40% of the general adult population worldwide [16]. MASLD considers factors ranging from abnormal accumulation of hepatic lipid droplets to metabolic alterations as important determinants for the diagnosis of the disease, highlighting the presence of type 2 diabetes or prediabetes, overweight/obesity, elevated blood pressure, higher triglyceride (TG) concentrations, or low high-density lipoprotein cholesterol (HDL-C) [3]. In MASLD, dyslipidemia is typically characterized by lower HDL-C and higher TG levels, and increased small, dense low-density lipoprotein particles, which are important risk factors for CVD [17, 18]. 

Previous studies [19, 20] on the association between MASLD and serum Lp(a) levels have yielded inconsistent or contradictory results, possibly due to differences in study design, population examined, and diagnostic criteria, which limit conclusions about this association. To our knowledge, no published studies have examined the association between MASLD and Lp(a) levels stratified by sex and menopausal status.

Therefore, we aimed to analyse sex- and menopausal status-specific associations between MASLD and circulating Lp(a) concentrations in two large population-based cohort studies, the Study of Health in Pomerania (SHIP) and the United Kingdom (UK) Biobank.

## Methods

### Study population

#### SHIP-START-0

The Study of Health in Pomerania is a population-based prospective cohort study conducted in the Northeast of Germany. The analysis presented here is based on data from the baseline SHIP-START-0. The study design has been described elsewhere [21–23]. Briefly, 4307 participants (2192 females, 50.9%; corresponding to a final response rate of 68.8%) aged 20 to 81 years took part in the baseline assessment conducted between 1997 and 2001. From this study population, we excluded individuals who reported cirrhosis (*n* = 17), individuals with steatotic liver disease (SLD) but without metabolic dysfunction-associated conditions (*n* = 13), and those with missing values for MASLD, serum alanine aminotransferase (ALT), aspartate aminotransferase (AST), gamma-glutamyltransferase (GGT), Lp(a), or any of the covariates (*n* = 452). The final analytical sample included 3825 individuals (1961 females; 51.3%) aged 32 to 70 years (Figure S1).

The study was approved by the Ethics Committee of the University of Greifswald. All participants provided written informed consent before enrolment, and the study was conducted in accordance with the principles of the Declaration of Helsinki [21]. 

### UK Biobank

The UK Biobank study is a large-scale, prospective, observational cohort study [24–27]. Briefly, 502,398 participants (273,317 females, 54.4%; corresponding to a final response rate of 5.50%) aged 37 to 73 years took part in the baseline assessment conducted between 2006 and 2010. Of these, 40,518 participants (21,158 females, 52.2%) aged 40 to 70 years who were eligible and willing to undergo a whole-body magnetic resonance imaging (MRI) scan participated in the liver MRI examination between 2014 and 2018. From this study population, we excluded individuals with missing Lp(a) values (*n* = 10,049), individuals with SLD but without metabolic dysfunction-associated conditions (*n* = 207), and those with missing values for MASLD, serum liver enzymes, or any of the covariates (*n* = 1758). The final analytical sample included 28,504 individuals (14,926 females; 52.4%) aged 40 to 62 years (Figure S2).

The study was conducted using the UK Biobank resource under approved application number 1,002,466. The UK Biobank scientific protocol and operational procedures were reviewed and approved on 17th June 2011by the North West MultiCentre Research Ethics Committee (reference: 11/NW/0382), extended on 13th May 2016 (reference: 16/NW/0274), and extended again on 18th June 2021 (reference: 21/NW/0157) in the UK [24]. All participants provided written informed consent before enrolment in the study, which was conducted in accordance with the principles of the Declaration of Helsinki [24]. 

### MASLD diagnostic criteria

In SHIP-START-0 and UK Biobank databases, MASLD diagnosis was based on the presence of SLD, as detected by liver ultrasonography (SHIP-START-0) or MRI (UK Biobank), with at least one concomitant metabolic dysfunction. These metabolic dysfunctions included body mass index (BMI) ≥ 25 kg/m² or waist circumference (WC) > 94 cm (males) and 80 cm (females) or ethnicity adjusted equivalent; fasting glucose ≥ 5.6 mmol/L (100 mg/dl) or 2-hour post-load glucose levels ≥ 7.8 mmol/L (≥ 140 mg/dl) or haemoglobin A1c ≥ 5.7% (39 mmol/L) or type 2 diabetes or current use of glucose-lowering medications; blood pressure ≥ 140/90 mmHg or current use of blood pressure-lowering medications; TG  ≥ 1.70 mmol/L (150 mg/dl) or current use of lipid-lowering medications; HDL-C  ≤ 1.0 mmol/L (40 mg/dl) (males) and ≤ 1.3 mmol/L (50 mg/dl) (females) or current use of lipid-lowering medications.

### Liver examination

#### SHIP-START-0

Liver ultrasound examinations were performed by trained physicians using a 7.5 MHz transducer and a high-resolution instrument (Vingmed VST Gateway, Santa Clara, CA) as described previously [28]. The sonographers were blinded to the participants’ clinical and laboratory characteristics. The presence of an ultrasonographically “bright liver” with clear contrast between hepatic and renal parenchyma was interpreted as ultrasonographic evidence for SLD [29]. 

### UK Biobank

Participants underwent MRI at the UK Biobank Imaging Centre in Cheadle, UK, using a Siemens Magnetom Aera 1.5T scanner (Siemens Healthineers, Erlangen, Germany) [30–32]. To acquire the data, a shortened modified look locker inversion (ShMOLLI) and a multiecho spoiled gradient-echo sequence were used [30–32]. 

A multiecho spoiled gradient-echo chemical-shift-encoded acquisition was used to generate proton density fat fraction (PDFF) maps of the liver. Image data were analysed, blinded to all other subject data, using Liver MultiScan Discover 4.0 software from Perspectum Diagnostics (UK) [30–32]. 

Liver fat content (LFC) was determined using PDFF values as reported previously [30]. Based on this, we categorized MASLD severity by using LFC values. Individuals with 0% to < 5% LFC were considered not to have MASLD, those with 5% to < 10% LFC were considered to have mild MASLD, those with 10% to < 20% were considered to have moderate MASLD, and those with ≥ 20% were considered to have severe MASLD.

### Laboratory measurements

#### SHIP-START-0

Serum ALT, AST, and GGT concentrations were measured photometrically using Hitachi 704 and 171 (Roche Diagnostics, Mannheim, Germany) [33]. 

Serum Lp(a) concentrations were measured by an immuno-luminometric assay using two polyclonal antibodies against apolipoprotein(a) on a Magic Lite Analyzer II (Ciba Corning, Fernwald, Germany) [14, 34]. 

Serum total cholesterol (TC), low-density lipoprotein cholesterol (LDL-C), and HDL-C concentrations were measured photometrically using a Hitachi 704 (Roche Diagnostics, Mannheim, Germany) [35, 36]. Serum TG concentrations were determined enzymatically using Roche Diagnostics reagents (Hitachi 717, Roche Diagnostics, Mannheim, Germany) [14, 35]. The triglycerides to high-density lipoprotein cholesterol (TG / HDL-C) ratio was calculated as TG divided by HDL-C [37, 38]. 

### UK Biobank

Serum ALT, AST, and GGT concentrations were analysed using an enzymatic method on a Beckman Coulter AU5800 (Beckman Coulter, UK, Ltd) [39]. 

Serum Lp(a) concentrations were measured by immuno-turbidimetric analysis on a Beckman Coulter AU5800 (Randox Biosciences, UK).

Serum TC and TG levels were measured by enzymatic analysis on a Beckman Coulter AU5800 (Beckman Coulter, UK, Ltd). Serum LDL-C levels were measured directly by enzymatic protective selection analysis on a Beckman Coulter AU5800 (Beckman Coulter, UK, Ltd). Serum HDL-C concentrations were measured by enzyme immuno-inhibition analysis on a Beckman Coulter AU5800 (Beckman Coulter, UK, Ltd). The TG / HDL-C ratio was also calculated [37, 38]. 

### Statistical analysis

To characterize the study sample, data were presented as medians (25th; 75th percentiles) for continuous variables and as percentages for categorical variables. Samples were stratified by MASLD status within each study, namely the SHIP-START-0 and UK Biobank cohorts.

In SHIP-START-0 and UK Biobank analyses, serum Lp(a) concentrations were logarithmically transformed because the residuals of linear regression models did not follow a normal distribution when the untransformed Lp(a) variable was used. Results of multivariable linear regression models were presented as geometric mean (GM) ratios with 95% confidence intervals (95% CI).

In SHIP-START-0 cohort, associations of MASLD, ALT, AST, and GGT levels with Lp(a) concentrations were assessed using linear regression models adjusted for age, haemoglobin A1c, glucose-lowering medication use, hypertension, body mass index, smoking, and daily alcohol consumption.

In UK Biobank cohort, associations of MASLD, MASLD severity (mild, moderate, and severe), MRI-measured LFC, and ALT, AST, and GGT levels with Lp(a) concentrations were assessed using linear regression analyses adjusted for the same covariates as in SHIP-START-0.

To assess whether the associations between liver transaminases or liver fat content and Lp(a) were linear, we tested fractional polynomials in our regression models using the mfp command. This approach systematically evaluates a pre-specified set of power transformations and selects the functional form that best fits the data based on the deviance, using a closed-test procedure. No evidence of non-linear associations was observed; therefore, a linear specification was retained in all models and figures.

Finally, we conducted sensitivity analyses to assess the association between changes in the TG / HDL-C ratio (exposure), i.e., a marker of insulin resistance [37, 38], and changes in Lp(a) concentrations (outcome). We determined the changes of both parameters by subtracting follow-up values (2010–2013) from baseline values (2006–2010). Associations were assessed using linear regression models adjusted for baseline and follow-up age, hypertension, body mass index, smoking, and daily alcohol consumption, after excluding individuals who were using glucose-lowering medications at baseline and/or follow-up examinations. For primary analyses, we decided to analyse SHIP-START-0 and UK Biobank cohorts separately to avoid inappropriate pooling of non-harmonized measurements which could have resulted in bias and compromised interpretability. On the other hand, in sensitivity analyses, we calculated meta-analysed estimates across both cohorts (random-effects models where appropriate) assessing the associations of MASLD, ALT, AST, and GGT levels with Lp(a) concentrations using linear regression models adjusted for age, haemoglobin A1c, glucose-lowering medication use, hypertension, body mass index, smoking, and daily alcohol consumption.

A two-sided p-value of less than 0.05 was considered statistically significant. All calculations were performed using Stata 19.5 (Stata Corporation, College Station, TX, USA).

## Results

### Description of the study populations

#### SHIP-START-0

In SHIP-START-0 cohort, 1102 (28.8%) of the 3825 participants were diagnosed with MASLD; 416 (10.9%) were females, and 686 (17.9%) were males (Table 1).

**Table 1:** Characteristics of the SHIP-START-0 population stratified by sex and metabolic dysfunction-associated steatotic liver disease (MASLD) (*n* = 3825)

Parameters	Females		Males	
	Without MASLD	With MASLD	Without MASLD	With MASLD
*N* (%)	1545 (40.4)	416 (10.9)	1178 (30.8)	686 (17.9)
Age (years)	44 (33; 58)	61 (52; 70)	46 (32; 63)	56 (45; 66)
Alanine aminotransferase (µkatal/L)	0.29 (0.23; 0.38)	0.42 (0.34; 0.59)	0.42 (0.32; 0.57)	0.64 (0.45; 0.91)
Aspartate aminotransferase (µkatal/L)	0.29 (0.25; 0.34)	0.33 (0.28; 0.41)	0.35 (0.30; 0.41)	0.41 (0.34; 0.54)
Gamma-glutamyltransferase (µkatal/L)	0.24 (0.18; 0.34)	0.35 (0.27; 0.54)	0.38 (0.27; 0.59)	0.65 (0.43; 1.04)
Lipoprotein(a) (mg/L)	96.0 (44.0; 271)	110 (48.0; 331)	96.0 (46.0; 275)	77.0 (37.0; 193)
Total cholesterol (mmol/L)	5.20 (4.50; 6.00)	5.70 (5.00; 6.40)	5.20 (4.50; 5.90)	5.50 (4.80; 6.20)
Low-density lipoprotein cholesterol (mmol/L)	3.00 (2.05; 3.70)	3.60 (3.00; 4.20)	3.20 (2.70; 3.80)	3.50 (2.90; 4.00)
High-density lipoprotein cholesterol (mmol/L)	1.45 (1.23; 1.71)	1.13 (0.92; 1.38)	1.15 (0.96; 1.38)	1.00 (0.84; 1.22)
Triglycerides (mmol/L)	1.20 (0.90; 1.70)	2.10 (1.50; 2.90)	1.50 (1.10; 2.20)	2.30 (1.60; 3.30)
Triglycerides / high-density lipoprotein cholesterol ratio	0.84 (0.56; 1.29)	1.80 (1.13; 3.02)	1.35 (0.84; 2.19)	2.34 (1.41; 3.71)
Dyslipidaemia (%)	31.7	68.3	47.7	69.5
Use of lipid-lowering medications (%)	4.20	13.7	8.00	11.2
Body mass index (kg/m^2^)	24.8 (22.3; 28.5)	30.7 (27.4; 34.5)	26.3 (23.8; 28.6)	29.3 (26.9; 32.0)
Waist circumference (cm)	78.0 (71.0; 87.0)	94.0 (86.0; 103)	92.0 (84.0; 99.0)	101 (95.0; 108)
Systolic blood pressure (mm Hg)	122 (112; 138)	141 (127; 154)	138 (126; 150)	145 (135; 158)
Diastolic blood pressure (mm Hg)	78 (72; 86)	84 (78; 90)	84 (76; 91)	88 (81; 96)
Hypertension (%)	32.9	72.8	53.0	77.7
Use of blood pressure-lowering medications (%)	18.5	51.4	23.0	38.6
Glucose (mmol/L)	5.00 (4.70.; 5.50)	5.6. (5.10.; 6.60)	5.30 (4.90; 5.70)	5.70 (5.20; 6.60)
Haemoglobin A1c (%)	5.10 (4.80; 5.50)	5.70 (5.20; 6.50)	5.3 (4.90; 5.70)	5.50 (5.00; 6.00)
Type 2 diabetes (%)	2.90	23.3	5.90	13.6
Use of glucose-lowering medications (%)	1.90	19.0	4.20	11.1
High-sensitivity C-reactive protein (mg/L)	1.20 (0.55; 2.85)	2.59 (1.26; 5.42)	1.02 (0.54; 2.27)	1.60 (0.86; 3.36)
Creatinine (µmol/L)	76.0 (70.0; 82.0)	77.0 (71.0; 86.0)	90.0 (84.0; 98.0)	90.0 (82.0; 99.0)
Estimated glomerular filtration rate (mL/min/1.73 m²)	82 (72; 93)	71 (63; 83)	88 (76; 98)	82 (72; 94)
Prevalent myocardial infarction (%)	0.50	3.10	3.10	5.00
Prevalent stroke (%)	0.80	0.70	2.70	2:80
Prevalent cardiovascular diseases (%)	1.30	3.60	5.40	6.80
Smoking status (%)				
Never smoker	46.5	60.6	23.6	17.9
Former smoker	23.2	21.9	40.2	52.6
Current smoker	30.4	17.5	36.2	29.4
Alcohol consumption (g/day)	3.90 (1.30; 8.40)	1.30 (0.00; 5.20)	10.2 (2.70; 22.6)	12.2 (3.80; 31.5)

Data is reported as medians, 25th ; 75th percentiles for continuous data or as absolute numbers and percentages for categorical data

Individuals with MASLD, regardless of sex, were older and had higher levels of serum liver enzymes (ALT, AST, and GGT), lipids (TC, LDL-C, and TG), glucose, haemoglobin A1c, hs-CRP, and greater adiposity measures (BMI and WC), whereas HDL-C levels were lower. Notably, serum Lp(a) levels differed by sex in individuals with MASLD. Females had higher Lp(a) concentrations than males. Serum creatinine levels were higher in females with MASLD, but similar between males with and without MASLD. Females and males with MASLD had higher systolic and diastolic blood pressure, were more likely to have a history of hypertension, type 2 diabetes, myocardial infarction, stroke, and CVD, and took blood pressure- and glucose-lowering medications more frequently. Females with MASLD were more frequently never smokers, whereas males with MASLD were more frequently former smokers. Females with MASLD consumed less alcohol than those without MASLD, whereas males with MASLD consumed more alcohol than those without MASLD (Table 1).

### UK Biobank

In UK Biobank cohort, 7608 (26.7%) of the 28,504 participants were diagnosed with MASLD; 3020 (10.6%) were females, and 4588 (16.1%) were males.

Among those with MASLD, 1774 (58.7%) were females with mild MASLD, 943 (31.2%) had moderate MASLD, and 303 (10.0%) had severe MASLD. Furthermore, 766 (60.3%) males had mild MASLD, 1424 (31.0%) had moderate MASLD, and 398 (8.70%) had severe MASLD (Table 2). Females with MASLD were older than males. As in the SHIP-START-0 sample, individuals with MASLD, of both sexes, had higher levels of serum liver enzymes (ALT, AST, and GGT), lipids (TC, LDL-C, and TG), glucose, haemoglobin A1c, hs-CRP, and greater adiposity measures (BMI and WC), whereas HDL-C levels were lower. As in the SHIP-START-0 sample, serum Lp(a) levels differed between sexes in individuals with MASLD. Females had higher Lp(a) concentrations than males. Contrary to the SHIP-START-0 sample, serum creatinine levels were similar between females with and without MASLD and higher in males with MASLD. Females and males with MASLD had higher systolic and diastolic blood pressure, were more likely to have a history of hypertension, type 2 diabetes, myocardial infarction, stroke, and CVD, and used more blood pressure- and glucose-lowering medications. Both females and males with MASLD reported never having smoked, but reported a higher frequency of alcohol consumption (Table 2).

**Table 2:** Characteristics of the UK Biobank population stratified by sex and metabolic dysfunction-associated steatotic liver disease (MASLD) (*n* = 28,504)

Parameters	Females		Males	
	Without MASLD	With MASLD	Without MASLD	With MASLD
*N* (%)	11,906 (41.8%)	3020 (10.6%)	8994 (31.6%)	4584 (16.1%)
Age (years)	54 (48; 60)	55 (50; 61)	57 (50; 62)	56 (50; 61)
Liver fat content by MRI (%)	2.30 (1.80; 3.00)	8.60 (6.30; 13.50)	2.80 (2.20; 3.50)	8.60 (6.30; 13.20)
MASLD severity (%)				
No	100	0.00	100	0.00
Mild	0.00	58.7	0.00	60.3
Moderate	0.00	31.2	0.00	31.0
Severe	0.00	10.0	0.00	8.70
Alanine aminotransferase (µkatal/L)	0.27 (0.22; 0.34)	0.35 (0.27; 0.46)	0.36 (0.29; 0.46)	0.48 (0.36; 0.64)
Aspartate aminotransferase (µkatal/L)	0.37 (0.33; 0.43)	0.39 (0.34; 0.46)	0.42 (0.37; 0.49)	0.46 (0.39; 0.54)
Gamma-glutamyltransferase (µkatal/L)	0.31 (0.24; 0.42)	0.42 (0.31; 0.60)	0.46 (0.35; 0.66)	0.63 (0.46; 0.93)
Lipoprotein(a) (mg/L)	86.0 (40.0; 245)	90.0 (40.0; 252)	87.0 (41.0; 265)	73.0 (35.0; 259)
Total cholesterol (mmol/L)	5.70 (5.10; 6.50)	5.90 (5.30; 6.70)	5.50 (4.80; 6.20)	5.60 (4.90; 6.40)
Low-density lipoprotein cholesterol (mmol/L)	3.50 (3.00; 4.00)	3.70 (3.20; 4.40)	3.50 (3.00; 4.10)	3.60 (3.10; 4.20)
High-density lipoprotein cholesterol (mmol/L)	1.65 (1.42; 1.90)	1.40 (1.21; 1.63)	1.31 (1.13; 1.53)	1.18 (1.02; 1.37)
Triglycerides (mmol/L)	1.10 (0.80; 1.50)	1.70 (1.30; 2.30)	1.50 (1.10; 2.10)	2.00 (1.50; 2.80)
Triglycerides / high-density lipoprotein cholesterol ratio	0.67 (0.48; 1.01)	1.21 (0.82; 1.79)	1.11 (0.72; 1.73)	1.71 (1.14; 2.58)
Dyslipidaemia (%)	42.7	59.9	52.8	69.5
Use of lipid-lowering medications (%)	5.40	11.7	14.4	21.6
Body mass index (kg/m^2^)	24.4 (22.4; 27.0)	28.7 (26.1; 32.1)	25.8 (23.9; 27.9)	28.4 (26.4; 30.9)
Waist circumference (cm)	78.0 (72.0; 85.0)	90.0 (83.0; 97.0)	91.0 (86.0; 98.0)	98.0 (93.0; 105)
Systolic blood pressure (mm Hg)	126 (116; 139)	133 (122; 146)	134 (124; 146)	138 (129; 149)
Diastolic blood pressure (mm Hg)	78 (72; 85)	82 (76; 89)	82 (76; 89)	85 (79; 92)
Hypertension (%)	30.9	50.1	47.2	62.5
Use of blood pressure-lowering medications (%)	8.10	18.6	14.5	22.9
Glucose (mmol/L)	4.80 (4.50; 5.20)	4.90 (4.60; 5.30)	4.90 (4.60; 5.20)	5.00 (4.60; 5.40)
Haemoglobin A1c (%)	5.29 (5.08; 5.50)	5.40 (5.17; 5.65)	5.30 (5.10; 5.51)	5.39 (5.15; 5.63)
Type 2 diabetes (%)	1.10	4.00	2.10	5.80
Use of glucose-lowering medications (%)	0.80	2.60	1.50	3.70
High-sensitivity C-reactive protein (mg/L)	0.90 (0.47; 1.86)	2.04 (1.08; 4.04)	0.90 (0.48; 1.72)	1.47 (0.81; 2.60)
Creatinine (µmol/L)	63.0 (57.0; 70.0)	63.0 (57.0; 70.0)	80.0 (73.0; 88.0)	81.0 (74.0; 88.0)
Estimated glomerular filtration rate (mL/min/1.73 m²)	95 (85; 102)	94 (84; 101)	93 (84; 99)	93 (84; 99)
Prevalent myocardial infarction (%)	1.20	2.50	5.40	6.30
Prevalent stroke (%)	0.70	0.80	1.70	1.80
Prevalent cardiovascular diseases (%)	1.80	3.20	6.90	8.00
Smoking (%)				
Never smoker	65.2	61.6	59.8	52.0
Former smoker	30.1	31.9	33.5	39.2
Current smoker	4.70	6.60	6.60	8.70
Alcohol status (%)				
Never drinker	2.90	4.80	1.80	1.70
Previous drinker	2.20	2.10	2.00	2.10
Current drinker	94.9	93.1	96.1	96.2

Data is reported as medians, 25th ; and 75th percentiles for continuous data or as absolute numbers and percentages for categorical data

### Associations of MASLD, LFC, serum liver enzymes, and the TG/HDL-C ratio with Lp(a) concentrations

#### SHIP-START-0

**Fig. 1:**
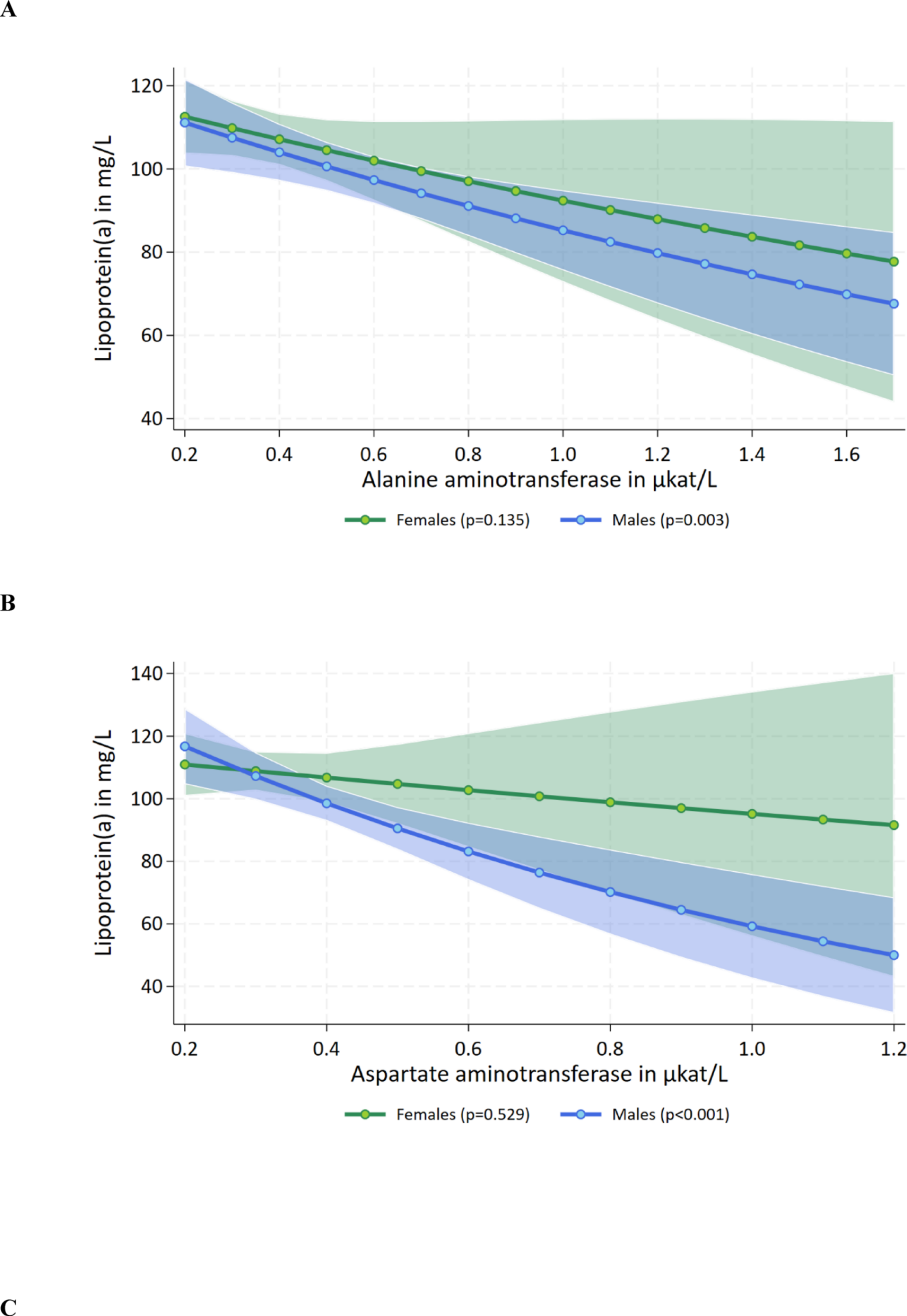

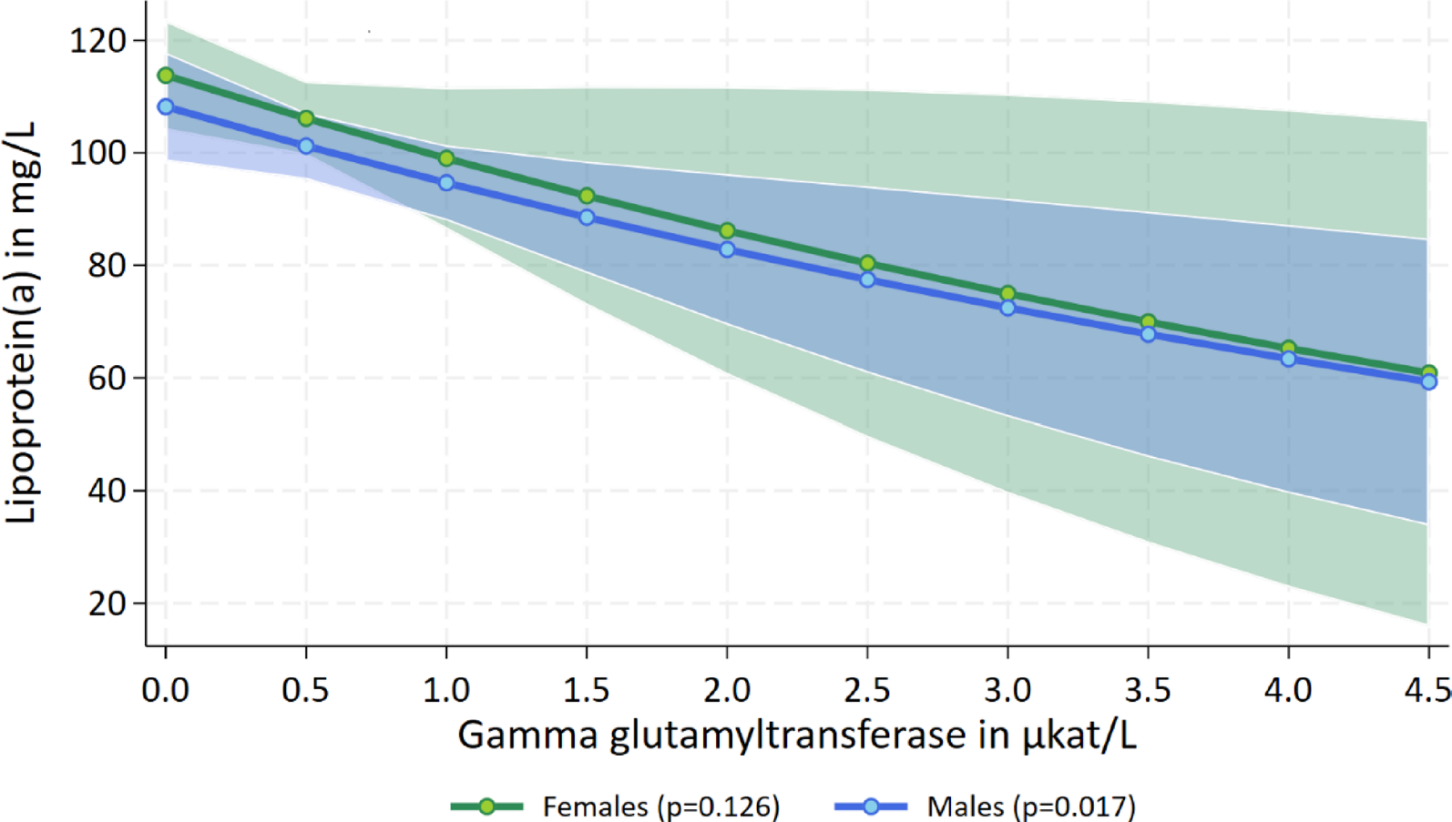
Adjusted* line (95% confidence interval) showing sex-specific associations of serum alanine aminotransferase (ALT) (**A**), aspartate aminotransferase (AST), (**B**), and gamma-glutamyltransferase (GGT), (**C**) levels with serum lipoprotein(a) concentrations in the SHIP-START-0. *Linear regression analysis adjusted for age, haemoglobin A1c, use of glucose-lowering medications, hypertension, body mass index, smoking status, and alcohol consumption

In multivariable linear regression models adjusting for age, body mass index, haemoglobin A1c, glucose-lowering medication use, hypertension, smoking, and alcohol consumption, we found that ultrasound-detected MASLD and higher ALT, AST, and GGT levels were associated with lower log-transformed Lp(a) concentrations in males. Conversely, no significant associations were observed in females. Specifically, males with MASLD had 24% lower log-transformed Lp(a) concentrations than their counterparts without MASLD. Moreover, 1 µkat/L higher ALT, AST, or GGT levels were associated with 28%, 57%, and 13% lower log-transformed Lp(a) concentrations, respectively (Table 3; Figs. 1A, B, and C). In contrast, after stratification by menopausal status, higher ALT levels were associated with lower log-transformed Lp(a) concentrations only in postmenopausal females. Specifically, 1 µkat/L higher ALT was associated with a 41% lower log-transformed Lp(a) concentration in postmenopausal females (Table 4).

**Table 3:** Adjusted* geometric mean (GM) ratios (95% confidence intervals [CI]) of associations between metabolic dysfunction-associated steatotic liver disease (MASLD), serum alanine aminotransferase (ALT), aspartate aminotransferase (AST), and gamma-glutamyltransferase (GGT) levels with log-transformed lipoprotein(a), stratified by sex in the SHIP-START-0 population (*n* = 3822)

Parameters	Females (*n* = 1959)	Males (*n* = 1863)
	GM ratios (95% CI); *p*-value	GM ratios (95% CI); *p*-value
MASLD (yes vs. no)	1.00 (0.80 to 1.18); *p* = 0.966	0.76 (0.67 to 0.87); p **< 0.001**
Alanine aminotransferase (µkat/L)	0.78 (0.56 to 1.08); *p* = 0.135	0.72 (0.58 to 0.89); p **= 0.003**
Aspartate aminotransferase (µkat/L)	0.83 (0.45 to 1.50); *p* = 0.529	0.43 (0.27 to 0.67); p **< 0.001**
Gamma-glutamyltransferase (µkat/L)	0.87 (0.73 to 1.04); *p* = 0.126	0.87 (0.78 to 0.98); p **= 0.017**

*Linear regression analysis adjusted for age, haemoglobin A1c, use of glucose-lowering medications, hypertension, body mass index, smoking status, and alcohol consumption

Data is expressed as %-change in log-transformed lipoprotein(a) levels by 1-unit increase in the respective exposure

**Table 4:** Adjusted* geometric mean (GM) ratios (95% confidence intervals [CI]) of associations between metabolic dysfunction-associated steatotic liver disease (MASLD), serum alanine aminotransferase (ALT), aspartate aminotransferase (AST), and gamma-glutamyltransferase (GGT) with log-transformed lipoprotein(a), stratified by females’ menopausal status in the SHIP-START-0 population (*n* = 1936)

Parameters	Premenopausal females (*n* = 1052)	Postmenopausal females (*n* = 884)
	GM ratios (95% CI); *p*-value	GM ratio (95% CI); *p*-value
MASLD (yes vs. no)	1.06 (0.79 to 1.42); *p* = 0.704	1.06 (0.87 to 1.29); *p* = 0.564
Alanine aminotransferase (µkat/L)	1.12 (0.70 to 1.79); *p* = 0.636	0.59 (0.37 to 0.94); p **= 0.027**
Aspartate aminotransferase (µkat/L)	1.23 (0.51 to 3.01); *p* = 0.645	0.66 (0.29 to 1.52); *p* = 0.327
Gamma-glutamyltransferase (µkat/L)	0.84 (0.60 to 1.17); *p* = 0.290	0.92 (0.74 to 1.14); *p* = 0.434

*Linear regression analysis adjusted for age, haemoglobin A1c, use of glucose-lowering medications, hypertension, body mass index, smoking status, and alcohol consumption

Data are expressed as %-change in log-transformed lipoprotein(a) levels by 1-unit increase in the respective exposure

### UK Biobank

**Fig. 2:**
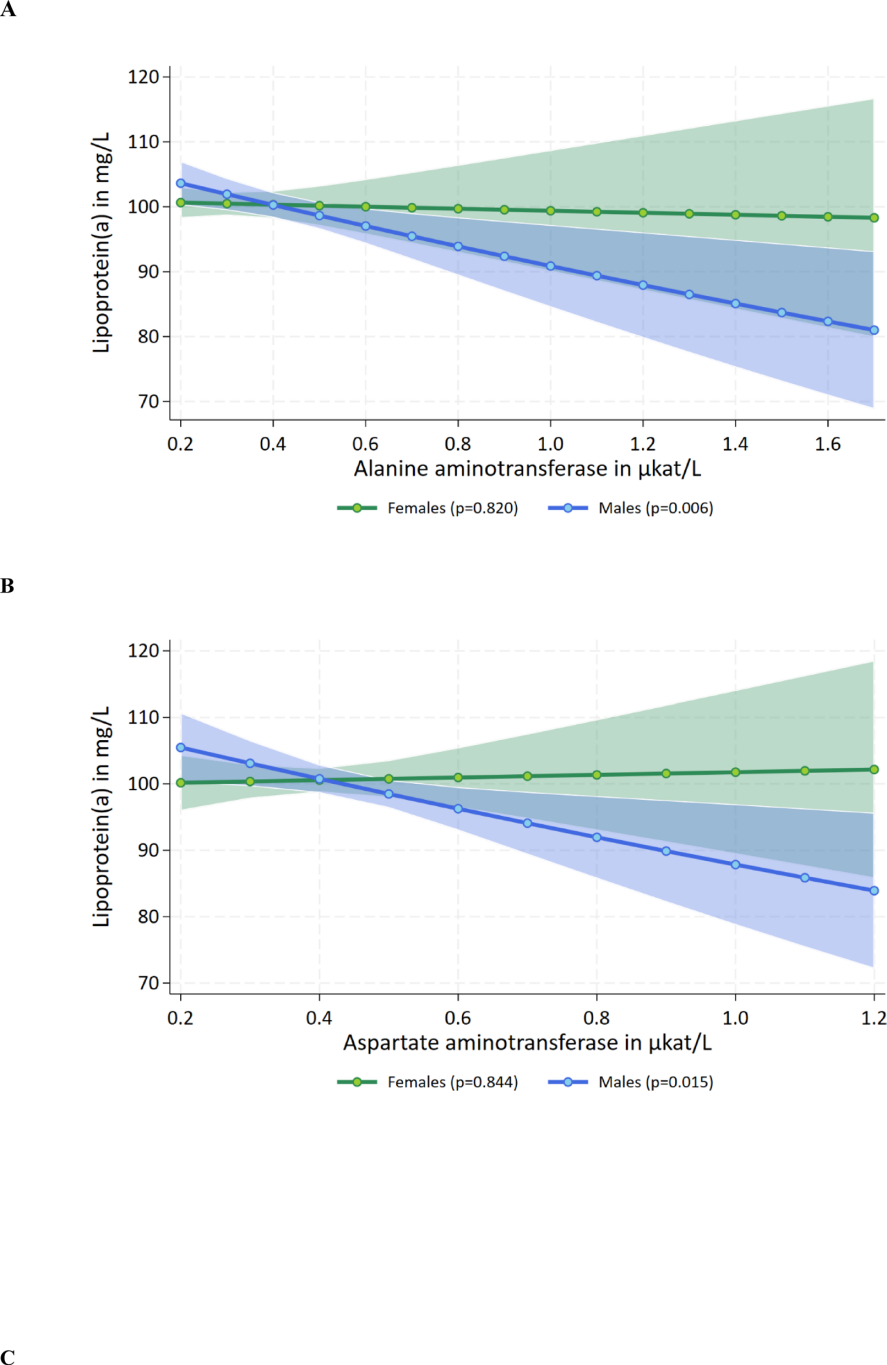

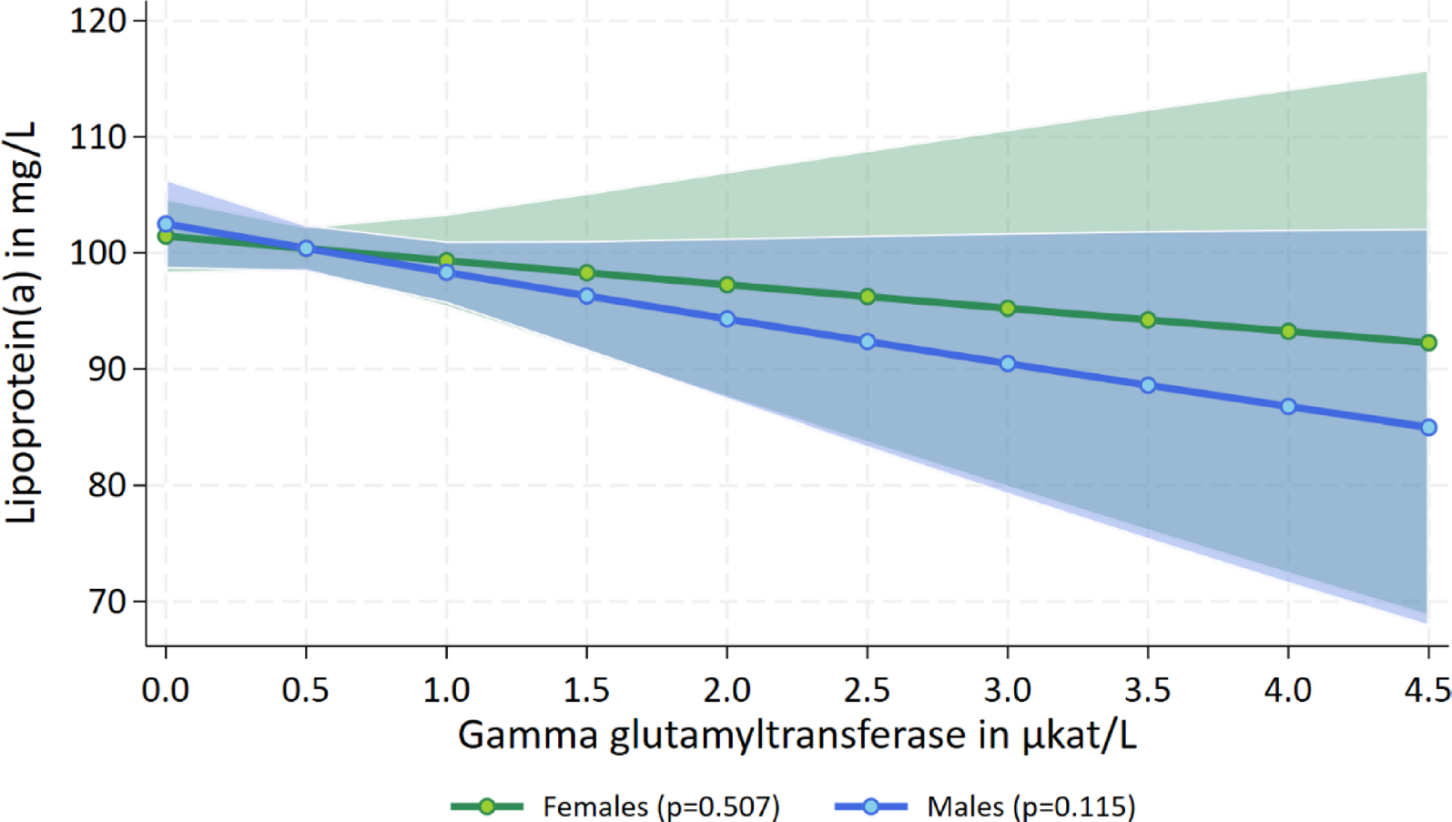
Adjusted* line (95% confidence interval) showing sex-specific associations of serum alanine aminotransferase (ALT) (**A**), aspartate aminotransferase (AST) (**B**), and gamma-glutamyltransferase (GGT) (**C**) levels with lipoprotein(a) concentrations in the UK Biobank. *Linear regression analysis adjusted for age, haemoglobin A1c, use of glucose-lowering medications, hypertension, body mass index, smoking status, and alcohol consumption

**Fig. 3:**
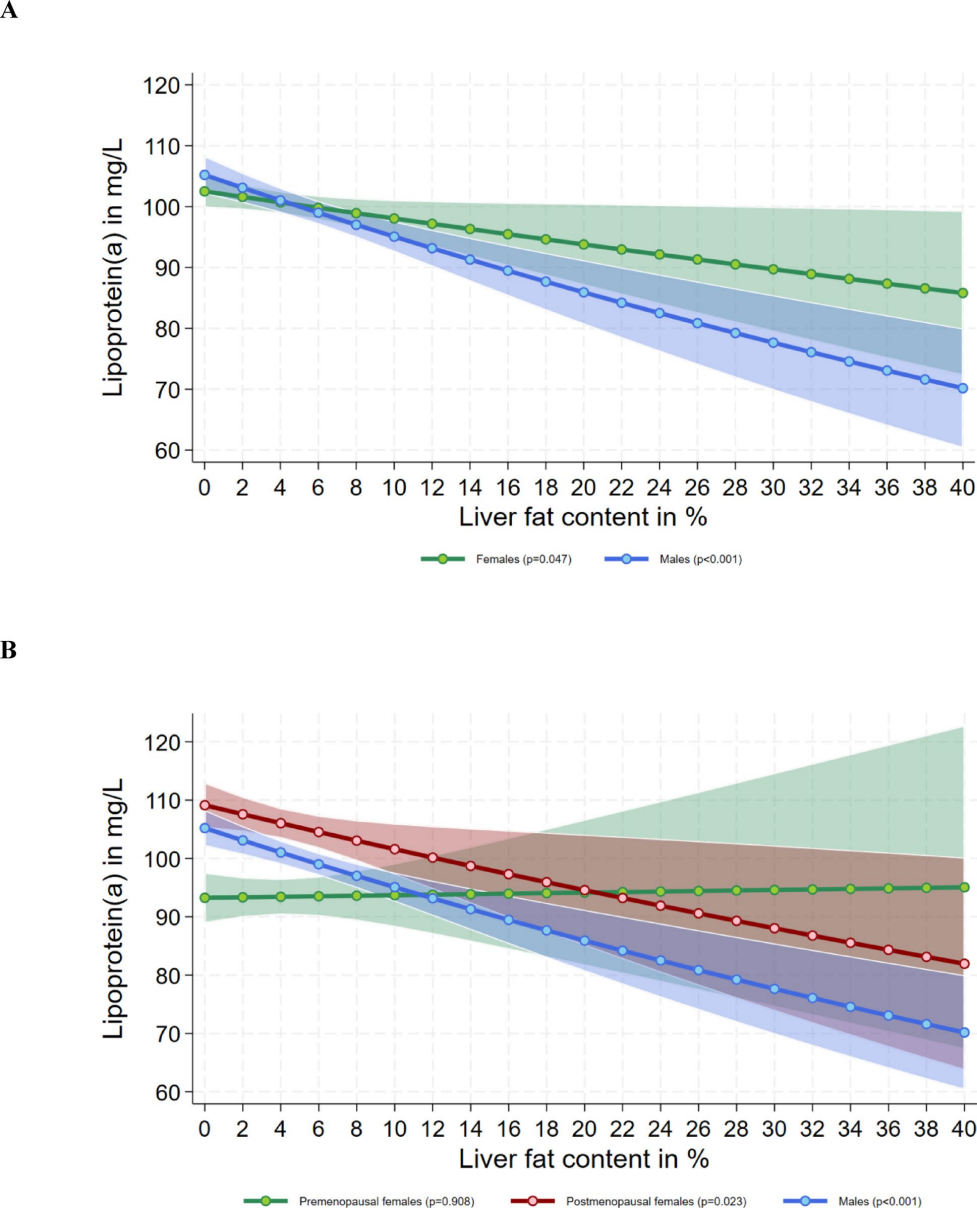
Adjusted* line (95% confidence interval) showing associations between liver fat content and serum lipoprotein(a) concentrations stratified by sex (**A**), and by sex and menopausal status (**B**) in the UK Biobank. *Linear regression adjusted for age, haemoglobin A1c, use of glucose-lowering medications, hypertension, body mass index, smoking status, and alcohol consumption

In multivariable linear regression models, only higher LFC was associated with lower log-transformed Lp(a) concentrations in females, whereas serum transaminases were not significantly associated. In males, MRI-detected MASLD, higher LFC, and higher ALT and AST levels (but not GGT) were associated with lower log-transformed Lp(a) concentrations. Specifically, in females, 1% higher LFC was associated with 2% lower log-transformed Lp(a) concentrations (Table 5; Fig. 2A, B, and C and 3 A). In males, individuals with MASLD had 9% lower log-transformed Lp(a) concentrations than those without MASLD. Moreover, males with moderate and severe MASLD had log-transformed Lp(a) concentrations 17% and 14% lower, respectively, than those without MASLD. Additionally, 1 µkat/L higher ALT and AST levels were associated with 15% and 20% lower log-transformed Lp(a) concentrations (Table 5; Fig. 2A, B, and C and 3 A). Notably, after stratification by menopausal status, postmenopausal females with severe MASLD had 21% lower log-transformed Lp(a) concentrations than postmenopausal females without MASLD, and 1% higher LFC was associated with 3% lower log-transformed Lp(a) concentrations. Conversely, no associations between any liver biomarker and log-transformed Lp(a) concentrations were observed in premenopausal females (Table 6; Fig. 3B).

**Table 5:** Adjusted* geometric mean (GM) ratios (95% confidence intervals [CI]) of the associations between metabolic dysfunction-associated steatotic liver disease (MASLD), liver fat content (LFC), serum alanine aminotransferase (ALT), aspartate aminotransferase (AST), and gamma-glutamyltransferase (GGT) with log-transformed lipoprotein(a), stratified by sex in the UK Biobank population (*n* = 28,504)

Parameters	Females (*n* = 14,926)	Males (*n* = 13,578)
	GM ratios (95% CI); *p*-value	GM ratios (95% CI); *p*-value
MASLD (yes vs. no)	0.99 (0.94 to 1.04); *p* = 0.568	0.91 (0.87 to 0.95); p **< 0.001**
Mild vs. no	1.00 (0.95 to 1.06); *p* = 0.908	0.96 (0.91 to 1.00); *p* = 0.073
Moderate vs. no	0.98 (0.91 to 1.06); *p* = 0.696	0.83 (0.78 to 0.89); p **< 0.001**
Severe vs. no	0.88 (0.77 to 1.00); *p* = 0.054	0.86 (0.77 to 0.96); p **= 0.010**
Liver fat content (%)	0.98 (0.96 to 1.00); p **= 0.047**	0.95 (0.93 to 0.97); p **< 0.001**
Alanine aminotransferase (µkat/L)	0.98 (0.86 to 1.13); *p* = 0.820	0.85 (0.75 to 0.95); p **= 0.006**
Aspartate aminotransferase (µkat/L)	1.01 (0.84 to 1.24); *p* = 0.844	0.80 (0.66 to 0.96); p **= 0.015**
Gamma-glutamyltransferase (µkat/L)	0.98 (0.92 to 1.04); *p* = 0.507	0.96 (0.91 to 1.01); *p* = 0.115

*Linear regression analysis adjusted for age, haemoglobin A1c, use of glucose-lowering medications, hypertension, body mass index, smoking status, and alcohol consumption

Data is expressed as %-change in log-transformed lipoprotein(a) levels by 1-unit increase in the respective exposure

**Table 6:** Adjusted* geometric mean (GM) ratios (95% confidence intervals [CI]) of the associations between metabolic dysfunction-associated steatotic liver disease (MASLD), liver fat content (LFC), serum alanine aminotransferase (ALT), aspartate aminotransferase (AST), and gamma-glutamyltransferase (GGT) with log-transformed lipoprotein(a), stratified by females` menopausal status in the UK Biobank population (*n* = 12,717)

Parameters	Premenopausal females (*n* = 4548)	Postmenopausal females (*n* = 8169)
	GM ratios (95% CI); *p*-value	GM ratios (95% CI); *p*-value
MASLD (yes vs. no)	1.01 (0.92 to 1.12); *p* = 0.762	0.97 (0.91 to 1.04); *p* = 0.444
Mild vs. no	1.01 (0.90 to 1.14); *p* = 0.785	0.99 (0.92 to 1.07); *p* = 0.895
Moderate vs. no	1.00 (0.86 to 1.17); *p* = 0.984	0.99 (0.89 to 1.10); *p* = 0.820
Severe vs. no	1.05 (0.82 to 1.33); *p* = 0.710	0.79 (0.66 to 0.95); p **= 0.015**
Liver fat content (%)	1.00 (0.96 to 1.04); *p* = 0.908	0.97 (0.94 to 0.99); p **= 0.023**
Alanine aminotransferase (µkat/L)	1.14 (0.86 to 1.51); *p* = 0.360	1.03 (0.86 to 1.23); *p* = 0.741
Aspartate aminotransferase (µkat/L)	1.09 (0.74 to 1.61); *p* = 0.671	1.01 (0.78 to 1.32); *p* = 0.912
Gamma-glutamyltransferase (µkat/L)	0.92 (0.80 to 1.05); *p* = 0.210	1.01 (0.94 to 1.10); *p* = 0.732

*Linear regression analysis adjusted for age, haemoglobin A1c, use of glucose-lowering medications, hypertension, body mass index, smoking status, and alcohol consumption

Data is expressed as %-change in log-transformed lipoprotein(a) levels by 1-unit increase in the respective exposure

In sensitivity analyses, we found a significant inverse association between changes in the TG / HDL-C ratio and changes in Lp(a) concentrations in both sexes. Notably, after stratification by menopausal status, postmenopausal females maintained a significant inverse association, whereas premenopausal females showed no association. Specifically, 1-unit increase in the TG / HDL-C ratio was associated with a decrease of 10.6 mg/L (95% CI 17.7–3.52; *p* = 0.003) in postmenopausal females and 5.07 mg/L in males (95% CI 8.40–1.74; *p* = 0.003), respectively. Likewise, in sensitivity analyses of the pooled SHIP-START-0 and UK Biobank cohorts we found, in general, similar results as the primary separated analyses.

## Discussion

To our knowledge, the present analysis, based on two large population-based cohort studies (SHIP-START-0 and UK Biobank) is the first to examine associations of MASLD, MRI-measured LFC, and liver enzymes with serum Lp(a) concentrations, stratified by sex and menopausal status. The prevalence of MASLD was 28.8% in SHIP-START-0 cohort and 26.7% in UK Biobank cohort. Our results show that MASLD and higher continuous LFC and ALT values were significantly associated with lower Lp(a) concentrations in postmenopausal females and males after adjustment for age, body mass index, haemoglobin A1c, glucose-lowering medication use, hypertension, smoking, and alcohol consumption. Although higher LFC was associated with lower Lp(a) concentrations in females, after stratification by menopausal status, this association was driven exclusively by postmenopausal females, whereas premenopausal females showed no association.

Although postmenopausal females may have Lp(a) levels that were approximately 17% higher than those of males [40], circulating Lp(a) levels are considered to be more than 90% determined by genetic factors and vary by race, but they are generally unaffected by conventional dietary strategies, such as a low-fat diet, moderate physical activity, or statin therapy [19]. Because apo(a) and apoB are assembled in the liver, any pathological condition that affects the normal function of hepatocytes may affect the production of these apolipoproteins, which are associated with changes in Lp(a) concentrations [41]. 

Some epidemiological studies [41–43] hypothesized that reduced Lp(a) levels might be the cause, rather than the consequence, of advanced MASLD. The detrimental effect of lower Lp(a) levels could be due to the retention of lipotoxic lipids in hepatocytes, as seen in heterozygous familial hypobetalipoproteinaemia. This may have translational relevance for the treatment of individuals with early-stage MASLD and elevated Lp(a) levels, because if the hypothesis is true, current under-development therapeutic strategies targeting Lp(a) reduction might worsen MASLD [44]. Contrary to this hypothesis, we found that longitudinal increases in the TG / HDL-C ratio, a marker of insulin resistance often accompanying the diagnosis of MASLD, were associated with lower Lp(a) concentrations. This finding suggests that changes in hepatocyte physiology following insulin resistance related to MASLD may lead to decreased hepatic Lp(a) production. Moreover, genetic predisposition to MASLD tended to be associated with lower Lp(a) levels, and liver damage was more likely the cause of reduced Lp(a) levels rather than a consequence [41]. This data provides reassurance about the long-term safety of Lp(a) lowering agents on liver health.

### In the context of the literature

A previous cross-sectional study [19] of 181 Japanese patients with MASLD (55.3% females), aged 20 to 79 years, found that individuals with steatohepatitis (MASH) had lower Lp(a) concentrations than patients with non-advanced fibrosis, independent of potential confounding factors.

Another cross-sectional study [20] of 151 middle-aged patients (67% females) with biopsy-confirmed MASLD also found that individuals with more advanced MASLD had 50% lower Lp(a) levels than those with less advanced liver disease.

A recent prospective study [42] of 56,168 Chinese patients with diagnosed MASLD (mean age 60.9 years, 45.9% females, followed for a median of 5 years) found that although advanced liver fibrosis was associated with lower Lp(a) concentrations, the incidence of major adverse CVD events was higher. These findings suggest that, in individuals with advanced liver disease, Lp(a) levels may underestimate the true CVD risk. In line with this study, in our sensitivity analyses (not shown), we found that in individuals with MASLD of both sexes, despite lower Lp(a) concentrations, circulating levels of LDL-C and hs-CRP were higher, which may result in a higher total cardiovascular risk, independent of circulating Lp(a) levels.

Another recent analysis [41] from the Liver-Bible cohort (859 participants with metabolic dysfunctions) and the Milan Biobank (DNA genotyping of 6963 individuals from the Italian population) found that genetic variation in the *LPA* gene was the strongest predictor of circulating Lp(a) levels, followed by liver stiffness measurement (as assessed by vibration-controlled transient elastography). Additionally, circulating Lp(a) levels, but not genetic predisposition, were inversely related to liver stiffness measurement, suggesting that MASLD severity may affect hepatic Lp(a) production. Again, in the Milan Biobank, genetically predicted higher Lp(a) levels tended to increase the risk of liver-related outcomes, whereas genetically predicted MASLD was associated with lower circulating Lp(a) levels [41]. Accordingly, the results of this study suggest that liver damage is more likely the cause of reduced Lp(a) levels rather than a consequence [41]. 

### Potential mechanisms for the observed associations

Current evidence suggests that hyperinsulinemia, rather than insulin resistance itself, suppresses hepatic Lp(a) production. MASLD is characterized by insulin resistance, leading to hepatic hyperinsulinemia [37]. An experimental study [45] using monkey hepatocyte cultures demonstrated that higher insulin levels reduced apo(a) synthesis in hepatocytes by suppressing mRNA expression, thereby lowering circulating Lp(a) concentrations. Although this experimental study involved hepatocytes from both female and male monkeys, it lacked sex-specific analyses [14]. Interestingly, a previous clinical study [46] using troglitazone, a glucose-lowering medication that decreases insulin levels, demonstrated an increase in Lp(a) concentrations, suggesting that hyperinsulinemia may have affected Lp(a) production. Again, there was no sex-specific analysis of these results [14]. Notably, type 1 diabetes, characterized by pancreatic insulin deficiency, is associated with higher Lp(a) concentrations that return to normal levels after starting insulin treatment [14, 45, 47]. 

Another contributing factor may be the direct loss and dysfunction of Lp(a)-producing hepatocytes following liver steatosis and fibrosis. Lp(a) is synthesized in hepatocytes by expressing the *LPA* gene [apo(a)] and assembling apo(a) with apoB. In a study [48] of individuals with biopsy-proven MASLD, both serum Lp(a) and hepatic *LPA* mRNA levels fell progressively as hepatic steatosis, steatohepatitis, and fibrosis worsened. *LPA* mRNA levels were closely associated with genes involved in hepatic LDL-C secretion, such as *APOB*, *APOA1*, and *MTTP*, indicating that as hepatocytes become steatotic and fibrotic, their capacity to transcribe *LPA* and to assemble and secrete lipoproteins is reduced due to direct impairment of liver function. Moreover, the same study [48] also showed that hepatic *LPA* mRNA expression was inversely associated with transforming growth factor beta (TGFβ) and collagen genes (*COL1A1*, *COL3A1*), classic markers and mediators of fibrogenesis. An experimental study [49] showed that TGFβ directly downregulates *LPA* transcription in hepatocytes. Thus, as MASLD progresses to fibrotic stages, TGFβ increases fibrogenic signalling pathways and decreases *LPA* gene expression, thereby decreasing apo(a) production and Lp(a) output [48]. Thus, MASLD appears to decrease hepatic Lp(a) production through a combination of coexistent hyperinsulinemia, reduced functional hepatocyte mass, and increased fibrogenic signalling pathways. These pathways can impair *LPA* mRNA transcription and reduce hepatic Lp(a) production in MASLD.

Importantly, our results showed that, while postmenopausal females showed an inverse association between MASLD and Lp(a) levels similar to that in males, premenopausal females showed no association, suggesting possible hormonal protection by oestrogens. In UK Biobank cohort, the overall prevalence of MASLD was 26.7%, with 20.2% among females and 33.8% among males. After stratification by menopausal status, the prevalence of MASLD was 20.3% in postmenopausal females and 16.9% in premenopausal females. Previous studies have shown that endogenous oestrogens can protect females by decreasing hepatic triglyceride production and fatty acid oxidation, thereby reducing oxidative stress, lipotoxicity, and inflammation in the liver, thus improving insulin resistance and, consequently, reducing hyperinsulinemia [50, 51]. 

### Clinical implications

The findings of this study may have important clinical implications. First, the significant association of MASLD and higher LFC with lower serum Lp(a) concentrations is not trivial. The assumption that Lp(a) concentrations are relatively stable over the lifetime should be questioned. Moreover, our societies are experiencing an obesity epidemic leading to several metabolic dysfunction conditions such as MASLD. Current and previous studies [14, 15] from our group, using four different population samples from North-eastern Germany [14, 15], Southern Germany [14], Berlin [15], and the United Kingdom, showed similar results regarding the association between metabolic dysfunction conditions and lower Lp(a) concentrations in postmenopausal females and males. Conversely, no associations were observed in premenopausal females. Interestingly, compared to individuals without type 2 diabetes, the relative increase in CVD mortality is much higher in females than in males with type 2 diabetes [14]. Oestrogen seems to have a “protective” effect against the development of insulin resistance in premenopausal females as compared with males [52]. It could be hypothesized that, paradoxically, the “protective” effect that stabilizes Lp(a) concentrations in premenopausal females under conditions of metabolic dysfunction may be responsible for the greater relative increase in CVD mortality in females compared to males. On the other hand, our analyses showed that postmenopausal females, which have a reduction in oestrogen, have a similar association of MASLD with lower Lp(a) concentrations as males.

Previous evidence [41] suggests that in patients with MASLD, metabolic syndrome, or type 2 diabetes, reduced Lp(a) concentrations may reflect impaired hepatic function, and that reduced Lp(a) levels alone may underestimate the true CVD risk in these patients [41]. Insulin resistance, hyperinsulinemia, or advanced liver fibrosis may act as independent or modifying risk factors, capable of maintaining or increasing CVD risk even when Lp(a) concentrations are reduced.

On the other hand, while pharmacotherapies targeting insulin resistance, hyperinsulinemia, and glucose levels may increase Lp(a) levels, their cardiometabolic benefits likely outweigh any increase in CVD risk associated with elevated Lp(a) concentrations, as evidenced by the fact that adequate glycaemic control improves patient survival [53, 54]. 

Finally, while several promising clinical trials are investigating Lp(a)-lowering medications [55, 56], these studies are still ongoing and have yet to provide results on their effects on CVD outcomes, and, more specifically, to clarify whether patients with associated metabolic dysfunction conditions will require more intensive treatment to reduce Lp(a) concentrations below the currently recommended cut-off values.

### Study limitations

A key limitation of this work is the observational nature of the analysed studies, which inherently restricts our ability to draw causal conclusions regarding the link between Lp(a) and MASLD. Although our findings reveal a clear correlation, they should be interpreted as hypothesis-generating rather than definitive proof of a metabolic cause-and-effect relationship. In the SHIP-START-0 cohort, ultrasonographic determination of MASLD and Lp(a) measurement were performed on the same day, while in the UK Biobank cohort, Lp(a) concentrations were measured around 8 years before the liver MRI examination. However, the significant inverse association that we observed between changes in the TG / HDL-C ratio and changes in Lp(a) concentrations suggests that the mechanisms potentially involved in our analyses were not affected by this temporal difference, as they result from a continuous process throughout participants’ lifetimes. Finally, despite adjusting for important confounders, we cannot exclude the influence of unmeasured or unknown factors. That said, it is important to note that our study has significant strengths, including its sizeable population-based design and the availability of multiple metabolic risk factors for adjustment.

## Conclusions

Our findings from two large community-based cohort studies indicate that MASLD and higher LFC and ALT values are significantly associated with lower serum Lp(a) concentrations in postmenopausal females and in males, even after adjusting for age, body mass index, hemoglobin A1c, glucose-lowering medication use, hypertension, smoking, and alcohol consumption. Further studies are needed to determine whether the CVD risk in patients with MASLD remains elevated even when Lp(a) concentrations are reduced, and whether these patients will require more intensive treatment to lower serum Lp(a) concentrations below the currently recommended cut-off values.

## Supplementary Information

Below is the link to the electronic supplementary material.

Supplementary Material 1

## Data Availability

The data from the SHIP study cannot be made publicly available due to the informed consent of the study participants, but it can be accessed through a data application form available at https://fvcm.med.uni-greifswald.de for researchers who meet the criteria for access to confidential data.The UK Biobank is a controlled-access dataset, and the trial data from this analysis cannot be shared, consistent with the original consent.

## Abbreviations

95% CI: 95% confidence intervals
ALT: alanine aminotransferase
AST: aspartate aminotransferase
Apo(a): apolipoprotein(a)
ApoB: apolipoprotein B
BMI: body mass index
CVD: cardiovascular diseases
CKD-EPI: Chronic Kidney Disease – Epidemiology Collaboration
DBP: diastolic blood pressure
eGFR: estimated glomerular filtration rate
GGT: gamma-glutamyltransferase
GM: geometric mean
HbA1c: haemoglobin A1c
HDL-C: high-density lipoprotein cholesterol
Hs-CRP: high-sensitivity C-reactive protein
LDL-C: low-density lipoprotein cholesterol
LFC: liver fat content
Lp(a)]: lipoprotein(a)
MASH: steatohepatitis
MASLD: metabolic dysfunction-associated steatotic liver disease
MRI: magnetic resonance imaging
NAFLD: non-alcoholic fatty liver disease
PDFF: proton density fat fraction
ROIs: regions of interest
SHIP: Study of Health in Pomerania
ShMOLLI: shortened modified look locker inversion
SBP: systolic blood pressure
SLD: steatotic liver disease
TC: total cholesterol
TG: triglycerides
TG/HDL-C ratio: triglycerides to high-density lipoprotein cholesterol ratio
TGFβ: transforming growth factor beta
UK: United Kingdom
WC: waist circumference
WHO: World Health Organization
