# Supplementary material for: Sex- and menopause-specific inverse associations between metabolic dysfunction–associated steatotic liver disease and serum lipoprotein(a) concentrations: evidence from SHIP and UK Biobank
Source: Cardiovasc Diabetol. 2026 Jul 12;25:203. doi: 10.1186/s12933-026-03289-8 (PMC13366839; doi:10.1186/s12933-026-03289-8)
Supplement: Supplementary file 1 — Supplementary Material 1 [file 12933_2026_3289_MOESM1_ESM.docx]

**Sex- and menopause-specific inverse associations between metabolic dysfunction–associated steatotic liver disease and lipoprotein(a) concentrations: Evidence from SHIP and UK Biobank**

| **Supplementary Material** | |
| --- | --- |
|  |  |
| **Expanded materials and methods** | **Page** |
| **Study population** | 2 |
| **Metabolic dysfunction-associated steatotic liver disease (MASLD) diagnostic criteria** | 2 |
| **Interview and medical examination** | 4 |
| **Liver examination** | 6 |
| **Laboratory measurements** | 8 |
| **Statistical analysis** | 11 |
| **References** | 12 |
| **Supplementary Figures** |  |
| **Figure S1:** Participants flow chart SHIP-START-0 study | 16 |
| **Figure S2:** Participants flow chart UK-Biobank study | 17 |
| **Supplementary Tables** |  |
| **Table S1:** Adjusted* geometric mean (GM) ratios (95% confidence intervals [CI]) of associations between metabolic dysfunction-associated steatotic liver disease (MASLD), serum alanine aminotransferase (ALT), aspartate aminotransferase (AST), and gamma-glutamyltransferase (GGT) levels with log-transformed lipoprotein(a), stratified by sex in the SHIP-START-0 (n = 3,822), UK Biobank (n = 28,504) and pooled (n = 32,326) populations. | 19 |
| **Table S2:** Adjusted* geometric mean (GM) ratios (95% confidence intervals [CI]) of associations between metabolic dysfunction-associated steatotic liver disease (MASLD), serum alanine aminotransferase (ALT), aspartate aminotransferase (AST), and gamma-glutamyltransferase (GGT) levels with log-transformed lipoprotein(a), stratified by females’ menopausal status in the SHIP-START-0 (n = 1,936), UK Biobank (n = 12,717) and pooled ( n = 14,653) populations. | 21 |

**Expanded methods**

**Study population**

**SHIP-START-0**

The Study of Health in Pomerania is a population-based prospective cohort study conducted in the Northeast of Germany. The analysis presented here is based on data from the baseline examination SHIP-START-0. The study design has been described in detail elsewhere.[1-3] In brief, a sample of the adult population aged 20 to 79 years was randomly selected using a multistage sampling procedure. The total population comprised 212,157 inhabitants. A total of 7,008 participants were sampled, with 292 persons of each sex in each of the twelve five-year age strata. The net sample (excluding migrated or deceased persons) comprised 6,265 eligible participants. Selected persons received a maximum of three written invitations. In cases of non-response, letters were followed by phone calls or home visits if phone contact was not possible. Of these, 4,307 participants (2,192 females, 50.9%; corresponding to a final response rate of 68.8%) aged 20 to 81 years took part in the baseline assessment conducted between 1997 and 2001. From this study population, we excluded individuals who reported cirrhosis (n = 17), individuals with SLD but without metabolic dysfunction-associated conditions (n = 13), and those with missing values for MASLD, serum alanine aminotransferase (ALT), aspartate aminotransferase (AST), gamma-glutamyltransferase (GGT), lipoprotein(a) (Lp[(a]), or any of the covariates (n = 452). The final analytical sample included 3,825 individuals (1,961 females; 51.3%) aged 32 to 70 years (**Figure S1**).

The study was approved by the ethics committee of the University of Greifswald. All participants provided written informed consent before enrolment in the study, which was conducted in accordance with the principles of the Declaration of Helsinki.[1]

**UK BIOBANK**

The UK Biobank study is a large-scale prospective observational cohort study.[4-7] Approximately 9.2 million individuals aged 40-69 years, recruited from the United Kingdom’s National Health Service (NHS) central registers and living in England, Wales, and Scotland, were invited to join the study. Of these, 502,398 participants (273,317 females, 54.4%; corresponding to a final response rate of 5.50%) aged 37 to 73 took part in the baseline assessment conducted between 2006 and 2010. Of these, 40,518 subjects (21,158 females, 52.2%) aged 40 to 70 years who were eligible and willing to undergo a whole-body MRI scan participated in the liver MRI examination between 2014 and 2018. From this study population, we excluded individuals with missing Lp(a) values (n = 10,049), individuals with SLD but without metabolic dysfunction-associated conditions (n = 207), and those with missing values for MASLD, ALT, AST, GGT, or any of the covariates (n = 1,758). The final analytical sample included 28,504 individuals (14,926 females; 52.4%) aged 40 to 62 years (**Figure S2**).

This research was conducted using the UK Biobank resource under approved application number 1002466. UK Biobank’s scientific protocol and operational procedures were reviewed and approved on 17th June 2011by the North West MultiCentre Research Ethics Committee (reference: 11/NW/0382) and extended on 13^th^ May 2016 (reference: 16/NW/0274) and on 18th June 2021(reference: 21/NW/0157) in the UK.[4] All participants provided written informed consent before enrolment in the study, which was conducted in accordance with the principles of the Declaration of Helsinki.[4]

**Metabolic dysfunction-associated steatotic liver disease (MASLD) diagnostic criteria**

In SHIP-START-0 and UK Biobank databases, MASLD diagnosis was based on the presence of steatotic liver disease (SLD), as detected by liver ultrasonography (SHIP-START-0) or magnetic resonance imaging (MRI) (UK Biobank), with at least one concomitant metabolic dysfunction. These metabolic dysfunctions included body mass index (BMI) ≥ 25 kg/m² or waist circumference (WC) > 94 cm (males) and 80 cm (females) or ethnicity adjusted equivalent; fasting glucose ≥ 5.6 mmol/L (100 mg/dl) or 2-hour post-load glucose levels ≥ 7.8 mmol/L (≥ 140 mg/dl) or haemoglobin A1c ≥ 5.7% (39 mmol/L) or type 2 diabetes or current use of glucose-lowering medications; blood pressure ≥ 140 / 90 mmHg or current use of blood pressure-lowering medications; triglycerides (TG) ≥ 1.70 mmol/L (150 mg/dl) or current use of lipid-lowering medications; high-density lipoprotein cholesterol (HDL-C) ≤ 1.0 mmol/L (40 mg/dl) (males) and ≤ 1.3 mmol/L (50 mg/dl) (females) or current use of lipid-lowering medications.

**Interview and medical examination**

**SHIP-START-0**

Information on age, sex, socioeconomic variables, smoking status (never, former, or current smoker), and medical history was collected through an interview conducted by trained and certified medical professionals. Alcohol consumption (in grams per day, g/d) was calculated by multiplying the frequency and amount of alcohol from beer, wine, and spirits, using a standard ethanol content of 4.8 percent (by volume) in beer, 11 percent (by volume) in wine, and 33 percent (by volume) in spirits for conversion.[8] Information on medication use was categorized according to the World Health Organization Anatomical Therapeutic Chemical Classification System.[2]

All participants underwent an extensive standardized physical examination. Body height (m), body weight (kg), and WC (cm) were measured according to World Health Organization (WHO) recommendations.[9] BMI was calculated as body weight (kg) divided by the square of body height (m²).[10] WC was measured to the nearest 0.1 cm using an inelastic tape measure midway between the lower rib margin and the iliac crest in the horizontal plane in individuals standing comfortably with weight distributed evenly on both feet.[11]

Systolic and diastolic blood pressure were measured three times after a five-minute rest period in the right arm of seated participants using a digital oscillometric blood pressure monitor (HEM-705CP, Omron Corporation, Tokyo, Japan). Measurements were separated by three-minute intervals. The average of the second and third measurements was calculated and used for the present analyses.

Hypertension was defined as systolic blood pressure (SBP) ≥ 140 mm Hg, and/or diastolic blood pressure (DBP) ≥ 90 mm Hg, or as the use of blood pressure-lowering medications defined by the ATC code (C02, C03, C07, C08, and C09).[12]

**UK BIOBANK**

Information on age, sex, socioeconomic variables, smoking status (never, former or current smoker), and medical history was collected by trained and certified medical professionals. Alcohol status was defined as never, former, or current drinker.

All participants underwent an extensive standardized physical examination. Body height (m), body weight (kg), and WC (cm) were measured according to World Health Organization (WHO) recommendations.[9] BMI was calculated as body weight (kg) divided by the square of body height (m²).[10] WC was measured to the nearest 0.1 cm using an inelastic tape measure midway between the lower rib margin and the iliac crest in the horizontal plane in individuals standing comfortably with weight distributed evenly on both feet.[11]

Systolic and diastolic blood pressure were measured twice after a one-minute rest period in the left arm of seated participants using a digital oscillometric blood pressure monitor (HEM-705 IT, Omron Corporation, Tokyo, Japan). Measurements were separated by a one-minute interval. The values from the first measurement were discarded, and those from the second were used in the analyses.

Hypertension was defined as SBP ≥ 140 mmHg and/or DBP ≥ 90 mmHg, or as the use of antihypertensive medications as defined by the ATC code (C02, C03, C07, C08, and C09).[12]

**Liver examination**

**SHIP-START-0**

Ultrasonographic examinations of the liver were performed by trained physicians using a 7.5 MHz transducer and a high-resolution instrument (Vingmed VST Gateway, Santa Clara, CA) as described previously.[13] The sonographers were blinded to the participants' clinical and laboratory characteristics. The presence of an ultrasonographically “bright liver” with clear contrast between hepatic and renal parenchyma was interpreted as ultrasonographic evidence of SLD.[14]

**UK BIOBANK**

## Participants underwent MRI at the UK Biobank Imaging Centre in Cheadle, UK, using a Siemens Magnetom Aera 1.5T scanner (Siemens Healthineers, Erlangen, Germany).[15-17] Participants with implanted defibrillators or metallic implants were excluded from the examination. To acquire the data, a shortened modified look locker inversion (ShMOLLI) and a multiecho spoiled gradient-echo sequence were used.[15-17] In both cases, a single transverse slice through the center of the liver, superior to the porta hepatis, was chosen to represent the liver. The acquisition was performed during expiratory apnea without contrast injection. The slice-based methodology has previously been shown to correlate well with histology and predict liver-related outcomes.[15-17]

## A multiecho spoiled gradient-echo chemical-shift-encoded acquisition was used to generate proton-density fat fraction (PDFF) maps of the liver. For PDFF, a three-point DIXON technique was applied to the complex data from the second, third, and fourth echoes. This technique accounts for magnetic field inhomogeneity and assumes that fat has a single-peak frequency. A 20-degree flip angle introduces some T1 bias, which has been shown to reduce PDFF values by a factor of 1.2 relative to a lower flip angle in an in-house implementation of the IDEAL methodology.[16]

Image data were analyzed, blinded to all other subject data, using Liver MultiScan Discover 4.0 software from Perspectum Diagnostics (UK).[15-17] For each PDFF image, three circular regions of interest (ROIs) with a 15-mm diameter were selected, and the mean pixel value within each ROI was calculated. ROIs were manually placed by a trained analyst to encompass a representative sample of the liver parenchyma, avoiding blood vessels, bile ducts, other organs, and MRI artifacts, if present.

Liver fat content (LFC) was determined using PDFF values as reported previously.[15] Based on this, we categorized MASLD severity by using LFC values. Individuals with 0% to < 5% LFC were considered not to have MASLD, those with 5% to < 10% LFC were considered to have mild MASLD, those with 10% to < 20% were considered to have moderate MASLD, and those with ≥ 20% were considered to have severe MASLD.

**Laboratory measurements**

**SHIP-START-0**

A non-fasting venous blood sample was obtained from all study participants between 07:00 a.m. and 04:00 p.m. using a vacuum collection tube while they were seated.[8]

Serum ALT, AST, and GGT concentrations were measured photometrically using Hitachi 704 and 171 (Roche Diagnostics, Mannheim, Germany).[18]

Serum Lp(a) concentrations were measured by an immuno-luminometric assay using two polyclonal antibodies against apolipoprotein(a) on a Magic Lite Analyzer II (Ciba Corning, Fernwald, Germany).[19, 20]

Serum total cholesterol (TC), low-density lipoprotein cholesterol (LDL-C), and HDL-C concentrations were measured photometrically using a Hitachi 704 (Roche Diagnostics, Mannheim, Germany).[21, 22] Serum triglycerides (TG) concentrations were determined enzymatically using Roche Diagnostics reagents (Hitachi 717, Roche Diagnostics, Mannheim, Germany).[20, 21] The triglycerides to high-density lipoprotein cholesterol (TG / HDL-C) ratio was calculated as TG divided by HDL-C.[23, 24] Dyslipidaemia was diagnosed based on serum cholesterol ≥ 6.2 mmol/L and/or LDL-C ≥ 4.1 mmol/L and/or total cholesterol / HDL-C ratio ≥ 5.0 and /or self-reported use of any lipid-lowering medication classified by the ATC code C10.[20, 25]

Glucose concentrations were determined enzymatically using Roche Diagnostics reagents (Hitachi 717, Roche Diagnostics, Mannheim, Germany).[20, 26] Haemoglobin A1c concentrations were determined by high-performance liquid chromatography (Diamat, Bio-Rad Laboratories, Munich, Germany).[22] Type 2 diabetes was diagnosed based on self-reported information, HbA1c ≥ 6.5%, non-fasting glucose ≥ 11.1 mmol/L, or the use of glucose-lowering medications defined by the ATC code A10.[25]

High-sensitivity C-reactive protein (hs-CRP) levels were measured immunologically on a Behring Nephelometer II using commercially available reagents (Dade Behring, Eschborn, Germany)[22, 26]

Serum creatinine concentration was measured using a modified kinetic Jaffé method (Hitachi 717, Roche Diagnostics, Mannheim, Germany). The estimated glomerular filtration rate (eGFR) was calculated using the Chronic Kidney Disease – Epidemiology Collaboration (CKD-EPI) equation and expressed in mL/min/1.73 m²: eGFR = 142 × min (serum creatinine / κ)^α^ × max (serum creatinine / κ) ^ - 1.200 × 0.9938^age^ × 1.012 (if females), where K is 0.7 for females and 0.9 for males, α is -0.241 for females and -0.302 for males, min indicates the minimum of serum creatinine / κ or 1, and max indicates the maximum of serum creatinine / κ or 1.

All assays were performed by skilled technical personnel in accordance with the manufacturer’s recommendations. In addition, the laboratory participates in official quarterly German external proficiency testing programs[27]

**UK BIOBANK**

A non-fasting venous blood sample was obtained from all study participants after approximately 8 hours of fasting, using a vacuum collection tube.[6]

Serum ALT, AST, and GGT concentrations were analysed using an enzymatic method on a Beckman Coulter AU5800 (Beckman Coulter, UK, Ltd).[28]

Serum Lp(a) concentrations were measured by immuno-turbidimetric analysis on a Beckman Coulter AU5800 (Randox Biosciences, UK).

Serum TC and TG levels were measured by enzymatic analysis on a Beckman Coulter AU5800 (Beckman Coulter, UK, Ltd). Serum LDL-C levels were measured directly by enzymatic protective selection analysis on a Beckman Coulter AU5800 (Beckman Coulter, UK, Ltd). Serum HDL-C concentrations were measured by enzyme immuno-inhibition analysis on a Beckman Coulter AU5800 (Beckman Coulter, UK, Ltd). The TG / HDL-C ratio was also calculated.[23, 24] Dyslipidaemia was defined as serum cholesterol ≥ 6.2 mmol/L and/or LDL-C ≥ 4.1 mmol/L and/or total cholesterol/HDL-C ratio ≥ 5.0 and/or self-reported use of any cholesterol-lowering medication.

Glucose concentrations were determined by an enzymatic analysis on a Beckman Coulter AU5800 (Beckman Coulter, UK, Ltd). HbA1c concentrations were measured by high-performance liquid chromatography on a Variant II Turbo Hemoglobin Test System (Bio-Rad Laboratories, Inc., California, USA). Type 2 diabetes was defined as self-reported and/or HbA1c ≥ 6.5%, non-fasting glucose ≥ 11.1 mmol/L, or self-reported use of any glucose-lowering medication.

Hs-CRP levels were measured by immunoturbidimetric analysis on a Beckman Coulter AU5800 (Beckman Coulter, UK, Ltd).

Serum creatinine concentration was determined by an enzymatic analysis on a Beckman Coulter AU5800 (Beckman Coulter, UK, Ltd). The estimated glomerular filtration rate (eGFR) was calculated using the Chronic Kidney Disease – Epidemiology Collaboration (CKD-EPI) equation[29] and expressed in mL/min/1.73 m²: eGFR = 142 × min (serum creatinine / κ)^α^ X max (serum creatinine / κ) ^ - 1.200 X 0.9938^age^ X 1.012 (if females) where K is 0.7 for females and 0.9 for males, α is -0.241 for females and -0.302 for males, min indicates the minimum of serum creatinine / κ or 1, and max indicates the maximum of serum creatinine / κ or 1.

All assays were performed by skilled technical personnel in accordance with the manufacturer’s recommendations.

**Statistical analysis**

To characterize the study sample, data were presented as medians (25^th^; 75^th^ percentiles) for continuous variables and as percentages for categorical variables. Samples were stratified by MASLD status within each study, namely the SHIP-START-0 and UK Biobank.

In SHIP-START-0 and UK Biobank analyses, Lp(a) concentrations were logarithmically transformed because the residuals of linear regression models did not follow a normal distribution when the untransformed Lp(a) variable was used. The results of multivariable linear regression models were presented as geometric mean (GM) ratios with 95% confidence intervals (95% CI).

In SHIP-START-0, associations of MASLD, ALT, AST, and GGT levels with Lp(a) concentrations were assessed using linear regression models adjusted for age, haemoglobin A1c , glucose-lowering medication use, hypertension, body mass index, smoking, and daily alcohol consumption.

In UK Biobank, associations of MASLD, MASLD severity (mild, moderate, and severe), MRI-measured LFC, and ALT, AST, and GGT levels with Lp(a) concentrations were assessed using linear regression analyses adjusted for the same covariates as in SHIP-START-0.

Finally, we conducted longitudinal sensitivity analyses to assess the association between changes in the TG / HDL-C ratio (exposure), i.e., a marker of insulin resistance,[24] and changes in Lp(a) concentrations (outcome). We determined the changes of both parameters by subtracting follow-up values (2010-2013) from baseline values (2006-2010). Associations were assessed using linear regression models adjusted for baseline and follow-up age, hypertension, body mass index, smoking, and daily alcohol consumption, after excluding individuals who were using glucose-lowering medications at baseline and/or follow-up examinations.

A two-sided p-value of less than 0.05 was considered statistically significant. All calculations were performed using Stata 19.5 (Stata Corporation, College Station, TX, USA).

**References**

1. John U, Greiner B, Hensel E, Lüdemann J, Piek M, Sauer S, Adam C, Born G, Alte D, Greiser E *et al*: **Study of Health In Pomerania (SHIP): a health examination survey in an east German region: objectives and design**. *Sozial- und Praventivmedizin* 2001, **46**(3):186–194.

2. Völzke H, Alte D, Schmidt CO, Radke D, Lorbeer R, Friedrich N, Aumann N, Lau K, Piontek M, Born G *et al*: **Cohort profile: the study of health in Pomerania**. *International journal of epidemiology* 2011, **40**(2):294–307.

3. Völzke H, Schössow J, Schmidt CO, Jürgens C, Richter A, Werner A, Werner N, Radke D, Teumer A, Ittermann T *et al*: **Cohort Profile Update: The Study of Health in Pomerania (SHIP)**. *International journal of epidemiology* 2022, **51**(6):e372–e383.

4. Sudlow C, Gallacher J, Allen N, Beral V, Burton P, Danesh J, Downey P, Elliott P, Green J, Landray M *et al*: **UK biobank: an open access resource for identifying the causes of a wide range of complex diseases of middle and old age**. *PLoS medicine* 2015, **12**(3):e1001779.

5. Bycroft C, Freeman C, Petkova D, Band G, Elliott LT, Sharp K, Motyer A, Vukcevic D, Delaneau O, O'Connell J *et al*: **The UK Biobank resource with deep phenotyping and genomic data**. *Nature* 2018, **562**(7726):203–209.

6. Caleyachetty R, Littlejohns T, Lacey B, Bešević J, Conroy M, Collins R, Allen N: **United Kingdom Biobank (UK Biobank): JACC Focus Seminar 6/8**. *Journal of the American College of Cardiology* 2021, **78**(1):56–65.

7. Rentsch CT, Garfield V, Mathur R, Eastwood SV, Smeeth L, Chaturvedi N, Bhaskaran K: **Sex-specific risks for cardiovascular disease across the glycaemic spectrum: a population-based cohort study using the UK Biobank**. *The Lancet regional health Europe* 2023, **32**:100693.

8. Baumeister SE, Völzke H, Marschall P, John U, Schmidt CO, Flessa S, Alte D: **Impact of fatty liver disease on health care utilization and costs in a general population: a 5-year observation**. *Gastroenterology* 2008, **134**(1):85–94.

9. **<WHO_TRS_854.pdf>**.

10. **Executive Summary**. *Obesity Research* 2012, **6**(S2).

11. Nishida C, Ko GT, Kumanyika S: **Body fat distribution and noncommunicable diseases in populations: overview of the 2008 WHO Expert Consultation on Waist Circumference and Waist-Hip Ratio**. *European journal of clinical nutrition* 2010, **64**(1):2–5.

12. Subcommittee. WHO-ISoHG: **1999 World Health Organization-International Society of Hypertension Guidelines for the Management of Hypertension. Guidelines Subcommittee**. *Journal of hypertension* 1999, **17**(2):151–183.

13. Volzke H, Robinson DM, Kleine V, Deutscher R, Hoffmann W, Ludemann J, Schminke U, Kessler C, John U: **Hepatic steatosis is associated with an increased risk of carotid atherosclerosis**. *World journal of gastroenterology* 2005, **11**(12):1848–1853.

14. Bellentani S, Saccoccio G, Masutti F, Crocè LS, Brandi G, Sasso F, Cristanini G, Tiribelli C: **Prevalence of and risk factors for hepatic steatosis in Northern Italy**. *Annals of internal medicine* 2000, **132**(2):112–117.

15. Wilman HR, Kelly M, Garratt S, Matthews PM, Milanesi M, Herlihy A, Gyngell M, Neubauer S, Bell JD, Banerjee R *et al*: **Characterisation of liver fat in the UK Biobank cohort**. *PloS one* 2017, **12**(2):e0172921.

16. Mojtahed A, Kelly CJ, Herlihy AH, Kin S, Wilman HR, McKay A, Kelly M, Milanesi M, Neubauer S, Thomas EL *et al*: **Reference range of liver corrected T1 values in a population at low risk for fatty liver disease-a UK Biobank sub-study, with an appendix of interesting cases**. *Abdominal radiology (New York)* 2019, **44**(1):72–84.

17. Parisinos CA, Wilman HR, Thomas EL, Kelly M, Nicholls RC, McGonigle J, Neubauer S, Hingorani AD, Patel RS, Hemingway H *et al*: **Genome-wide and Mendelian randomisation studies of liver MRI yield insights into the pathogenesis of steatohepatitis**. *Journal of hepatology* 2020, **73**(2):241–251.

18. Völzke H, Alte D, Ittermann T, Schmidt CO, Rettig R, Mayerle J, Lowenfels AB, Lerch MM, Nauck M: **Subjects with sonographical hepatic steatosis should be excluded from studies to establish upper reference levels of serum transaminases**. *Liver international : official journal of the International Association for the Study of the Liver* 2011, **31**(7):985–993.

19. Keßler A, Schumacher M, Wood WG: **Immunoluminometric Assays for the Quantification of Apolipoproteins A-I, B, C-II, Apolipoprotein(a) and Lipoprotein(a)**. In*.*: Kooperation de Gruyter; 1994.

20. Markus MRP, Ittermann T, Schipf S, Bahls M, Nauck M, Völzke H, Santos RD, Peters A, Zeller T, Felix SB *et al*: **Association of sex-specific differences in lipoprotein(a) concentrations with cardiovascular mortality in individuals with type 2 diabetes mellitus**. *Cardiovascular diabetology* 2021, **20**(1):168.

21. Haring R, Feng YS, Moock J, Völzke H, Dörr M, Nauck M, Wallaschofski H, Kohlmann T: **Self-perceived quality of life predicts mortality risk better than a multi-biomarker panel, but the combination of both does best**. *BMC medical research methodology* 2011, **11**:103.

22. Schneider HJ, Wallaschofski H, Völzke H, Markus MR, Doerr M, Felix SB, Nauck M, Friedrich N: **Incremental effects of endocrine and metabolic biomarkers and abdominal obesity on cardiovascular mortality prediction**. *PloS one* 2012, **7**(3):e33084.

23. Colantoni A, Bucci T, Cocomello N, Angelico F, Ettorre E, Pastori D, Lip GYH, Del Ben M, Baratta F: **Lipid-based insulin-resistance markers predict cardiovascular events in metabolic dysfunction associated steatotic liver disease**. *Cardiovascular diabetology* 2024, **23**(1):175.

24. Liu L, Yu G, Ji X, Wang Y, He H: **Associations of six insulin resistance-related indices with the risk and progression of cardio-renal-metabolic multimorbidity: evidence from the UK biobank**. *Cardiovascular diabetology* 2025, **24**(1):377.

25. Markus MRP, Ittermann T, Mariño Coronado J, Schipf S, Bahls M, Könemann S, Chamling B, Völzke H, Damasceno NRT, Santos RD *et al*: **Low-density lipoprotein cholesterol, lipoprotein(a) and high-sensitivity C-reactive protein are independent predictors of cardiovascular events**. *European heart journal* 2025, **46**(39):3863–3874.

26. Friedrich N, Schneider HJ, Spielhagen C, Markus MR, Haring R, Grabe HJ, Buchfelder M, Wallaschofski H, Nauck M: **The association of serum prolactin concentration with inflammatory biomarkers - cross-sectional findings from the population-based Study of Health in Pomerania**. *Clinical endocrinology* 2011, **75**(4):561–566.

27. Haring R, Völzke H, Steveling A, Krebs A, Felix SB, Schöfl C, Dörr M, Nauck M, Wallaschofski H: **Low serum testosterone levels are associated with increased risk of mortality in a population-based cohort of men aged 20-79**. *European heart journal* 2010, **31**(12):1494–1501.

28. Sun X, Guo Z, Zhang Y, Liu Z, Xiong J, Cai M, Tan J, Lin Y, Yu Z, Du K *et al*: **Liver Function Biomarkers and Lung Cancer Risk: A Prospective Cohort Study in the UK Biobank**. *The clinical respiratory journal* 2024, **18**(12):e70042.

29. Inker LA, Eneanya ND, Coresh J, Tighiouart H, Wang D, Sang Y, Crews DC, Doria A, Estrella MM, Froissart M *et al*: **New Creatinine- and Cystatin C-Based Equations to Estimate GFR without Race**. *N Engl J Med* 2021, **385**(19):1737–1749.

**Supplementary Figures**

**Figure S1:** Participants flow chart SHIP-START-0 study.

SHIP-START-0 participants

(n = 4,307)

)

Abdominal magnetic resonance imaging (MRI)

(n = 4,307)

)

Exclusions (n = 482)

- Individuals with liver cirrhosis ​​ (n = 17)
- Individuals without risk factors for metabolic dysfunction-associated steatotic liver disease (MASLD) (n = 13)
- Individuals without values for MASLD, alanine aminotransferase (ALT), aspartate aminotransferase (AST), gamma-glutamyltransferase (GGT), lipoprotein(a) (Lp[a]) or any of the covariates (n = 452)

Complete data for analysis

(n = 3,825)

(n = 322,922)

**Figure S2:** Participants flow chart UK Biobank study.

UK Biobank participants

(n = 502,398)

)

Abdominal magnetic resonance imaging (MRI)

(n = 40,518)

)

Exclusions (n = 12,014)

- Individuals with absent lipoprotein(a) (Lp[a]) values ​​ (n = 10,049)
- Individuals without risk factors for metabolic dysfunction-associated steatotic liver disease (MASLD) (n = 207)
- Individuals without values for MASLD, alanine aminotransferase (ALT), aspartate aminotransferase (AST), gamma-glutamyltransferase (GGT), or any of the covariates who don’t have information on any other covariate (n = 1,758)

Complete data for analysis

(n = 28,504)

(n = 322,922)

**Supplementary Tables**

**Table S1:** Adjusted* geometric mean (GM) ratios (95% confidence intervals [CI]) of associations between metabolic dysfunction-associated steatotic liver disease (MASLD), serum alanine aminotransferase (ALT), aspartate aminotransferase (AST), and gamma-glutamyltransferase (GGT) levels with log-transformed lipoprotein(a), stratified by sex in the SHIP-START-0 (n = 3,822), UK Biobank (n = 28,504) and pooled (n = 32,326) populations.

|  | **Females** | | | **Males** | | |
| --- | --- | --- | --- | --- | --- | --- |
|  | **SHIP-START-0**  **(n = 1,959)** | **UK Biobank**  **(n = 14,926)** | **Pooled**  **(n = 16,885)** | **SHIP-START-0**  **(n = 1,863)** | **UK Biobank**  **(n = 13,578)** | **Pooled**  **(n = 15,441)** |
|  | **GM ratios (95% CI)**  **p-value** | | | | | |
| **Parameters** |  |  |  |  |  |  |
| **MASLD (yes vs. no)** | 1.00 (0.80 to 1.18)  p=0.966 | 0.99 (0.94 to 1.04)  p=0.568 | 0.99 (0.94 to 1.04)  p=0.706 | 0.76 (0.67 to 0.87)  p**<0.001** | 0.91 (0.87 to 0.95)  p**<0.001** | 0.85 (0.76 to 0.96)  **p=0.007** |
| **Alanine aminotransferase (µkat/L)** | 0.78 (0.56 to 1.08)  p=0.135 | 0.98 (0.86 to 1.13)  p=0.820 | 0.95 (0.84 to 1.12)  p=0.403 | 0.72 (0.58 to 0.89)  p**=0.003** | 0.85 (0.75 to 0.95)  p**=0.006** | 0.82 (0.74 to 0.96)  **p<0.001** |
| **Aspartate aminotransferase (µkat/L)** | 0.83 (0.45 to 1.50)  p=0.529 | 1.01 (0.84 to 1.24)  p=0.844 | 0.99 (0.82 to 1.19)  p=0.927 | 0.43 (0.27 to 0.67)  p**<0.001** | 0.80 (0.66 to 0.96)  p**=0.015** | 0.63 (0.42 to 0.95)  **p=0.027** |
| **Gamma-glutamyltransferase (µkat/L)** | 0.87 (0.73 to 1.04)  p=0.126 | 0.98 (0.92 to 1.04)  p=0.507 | 0.97 (0.91 to 1.03)  p=0.265 | 0.87 (0.78 to 0.98)  p**=0.017** | 0.96 (0.91 to 1.01)  p=0.115 | 0.95 (0.90 to 0.99)  **p=0.017** |

*Linear regression analysis adjusted for age, haemoglobin A1c, use of glucose-lowering medications, hypertension, body mass index, smoking status, and alcohol consumption.

Data is expressed as %-change in log-transformed lipoprotein(a) levels by 1-unit increase in the respective exposure.

For the pooled estimate, the study-specific estimates were combined using random-effects meta-analysis.

**Table S2:** Adjusted* geometric mean (GM) ratios (95% confidence intervals [CI]) of associations between metabolic dysfunction-associated steatotic liver disease (MASLD), serum alanine aminotransferase (ALT), aspartate aminotransferase (AST), and gamma-glutamyltransferase (GGT) levels with log-transformed lipoprotein(a), stratified by females’ menopausal status in the SHIP-START-0 (n = 1,936), UK Biobank (n = 12,717) and pooled (n = 14,653) populations.

|  | **Premenopausal females** | | | **Postmenopausal females** | | |
| --- | --- | --- | --- | --- | --- | --- |
|  | **SHIP-START-0**  **(n = 1,052)** | **UK Biobank**  **(n = 4,548)** | **Pooled**  **(n = 5,600)** | **SHIP-START-0**  **(n = 884)** | **UK Biobank**  **(n = 8,169)** | **Pooled**  **(n = 9,053)** |
|  | **GM ratios (95% CI)**  **p-value** | | | | | |
| **Parameters** |  |  |  |  |  |  |
| **MASLD (yes vs. no)** | 1.06 (0.79 to 1.42)  p=0.704 | 1.01 (0.92 to 1.12)  p=0.762 | 1.01 (0.93 to 1.11)  p=0.755 | 1.06 (0.87 to 1.29)  p=0.564 | 0.97 (0.91 to 1.04)  p=0.444 | 0.98 (0.92 to 1.04)  p=0.509 |
| **Alanine aminotransferase (µkat/L)** | 1.12 (0.70 to 1.79)  p=0.636 | 1.14 (0.86 to 1.51)  p=0.360 | 1.14 (0.89 to 1.44)  p=0.305 | 0.59 (0.37 to 0.94)  p**=0.027** | 1.03 (0.86 to 1.23)  p=0.741 | 0.86 (0.61 to 1.21)  p=0.384 |
| **Aspartate aminotransferase (µkat/L)** | 1.23 (0.51 to 3.01)  p=0.645 | 1.09 (0.74 to 1.61)  p=0.671 | 1.11 (0.78 to 1.59)  p=0.561 | 0.66 (0.29 to 1.52)  p=0.327 | 1.01 (0.78 to 1.32)  p=0.912 | 0.95 (0.68 to 1.31)  p=0.735 |
| **Gamma-glutamyltransferase (µkat/L)** | 0.84 (0.60 to 1.17)  p=0.290 | 0.92 (0.80 to 1.05)  p=0.210 | 0.91 (0.80 to 1.05)  p=0.134 | 0.92 (0.74 to 1.14)  p=0.434 | 1.01 (0.94 to 1.10)  p=0.732 | 1.00 (0.93 to 1.08)  P=0.980 |

*Linear regression analysis adjusted for age, haemoglobin A1c, use of glucose-lowering medications, hypertension, body mass index, smoking status, and alcohol consumption.

Data is expressed as %-change in log-transformed lipoprotein(a) levels by 1-unit increase in the respective exposure.

For the pooled estimate, the study-specific estimates were combined using random-effects meta-analysis.
